# Genetic Structure and Population History of the Zaisan Toad-Headed Agama (*Phrynocephalus melanurus*) Inferred from Mitochondrial DNA

**DOI:** 10.3390/ani14020209

**Published:** 2024-01-08

**Authors:** Daniya Ualiyeva, Jinlong Liu, Tatjana Dujsebayeva, Jun Li, Lili Tian, Bo Cai, Xiaomao Zeng, Xianguang Guo

**Affiliations:** 1 Chengdu Institute of Biology, Chinese Academy of Sciences, Chengdu 610041, China; daniya.2010@mail.ru (D.U.); liujl@cib.ac.cn (J.L.); tianll@cib.ac.cn (L.T.); caibo@cib.ac.cn (B.C.); 2 University of Chinese Academy of Sciences, Beijing 100049, China; 3 Laboratory of Ornithology and Herpetology, Institute of Zoology CS MES RK, 93 al-Farabi Avenue, Almaty 050060, Kazakhstan; tatjana.dujsebayeva@zool.kz; 4 College of Life Science and Technology, Xinjiang University, Urumqi 830046, China; lijuncherish@163.com

**Keywords:** agamid, aridification, colonization, mitochondrial DNA, Northwest China, phylogeography, species distribution models

## Abstract

**Simple Summary:**

The effects of Quaternary climatic oscillations on lineage diversification and demography of organisms in drylands have drawn much attention recently. However, little is still known about the processes that shaped the species’ spatial genetic structure in areas such as the arid Central Asia, particularly for animals in Northwest China. Here, we investigated the genetic structure and population dynamics of the Zaisan toad-headed agama (*Phrynocephalus melanurus*) by combining mtDNA phylogeography and species distribution models (SDMs) with range-wide sampling for the first time. Phylogenetic analyses recovered two main Clades, with one from Dzungar and Alakol basins being geographically sub-structured into several groups. Lineage diversification took place in the Pleistocene, coinciding with the drastic aridification caused by Quaternary climatic transitions and drastic activity of the Tianshan Mountains. Moreover, populations of the Dzungar Basin experienced the past expansion and parapatric divergence contributed by isolation-by-distance. SDMs unveiled the species range dynamics since the late Pleistocene, showing expansion in interglacial, and contraction during last glacial maximum and late Holocene periods. Future distribution projections demonstrated drastic habitat loss, suggesting the significance of conservation effort. Our findings highlight the significance of combining genetic approaches with environmental data when evaluating the effects of Pleistocene climatic oscillations.

**Abstract:**

The agamid lizard *Phrynocephalus melanurus* is restricted to Northwest China (Dzungar Basin) and the adjacent Eastern Kazakhstan (Zaisan and Alakol basins). To elucidate the phylogeography of *P. melanurus*, we obtained the mitochondrial DNA *COI* segments of 175 sampled lizards from 44 localities across the whole distribution. Phylogenetic analyses revealed two main Clades comprising five geographically structured lineages (I, IIa, IIb1, IIb2, and IIb3) that fit an isolation-by-distance (IBD) model. The divergence from the most recent common ancestor was dated to ~1.87 million years ago (Ma). Demographic analyses demonstrated lineage-specific response to past climate change: stable population for Clade I, Subclade IIb1; past population expansion for IIb3 since 0.18 Ma, respectively. Bayesian phylogeographic diffusion analyses detected initial spreading at the Saur Mount vicinity, approximately 1.8 Ma. Historical species distribution model (SDM) projected expansion of the suitable habitat in the last interglacial and shift and contraction in the last glacial maximum and Holocene epochs. The SDM predicted a drastic reduction in suitable area throughout the range as a response to future climate change. Our findings suggest that the evolution of *P. melanurus* followed a parapatric divergence with subsequent dispersal and adaptation to cold and dry environments during the Quaternary. Overall, this work improves our understanding of the lineage diversification and population dynamics of *P. melanurus*, providing further insights into the evolutionary processes that occurred in Northwest China and adjacent Eastern Kazakhstan.

## 1. Introduction

Historical geological events and climate changes, coupled with the substantial environmental heterogeneity, have shaped the phylogeography of organisms across the globe. This complex interplay may have significantly influenced the processes of diversification and speciation of local biota in drylands [1,2]. It has been recognized that the climate fluctuations during the Quaternary have greatly impacted the distribution of arid-adapted organisms [3,4,5,6]. Thus, many species adapted to the cold and dry environments have recently attracted much attention in the field of biogeography and phylogeography [7,8,9,10,11]. 

**Figure 1:**
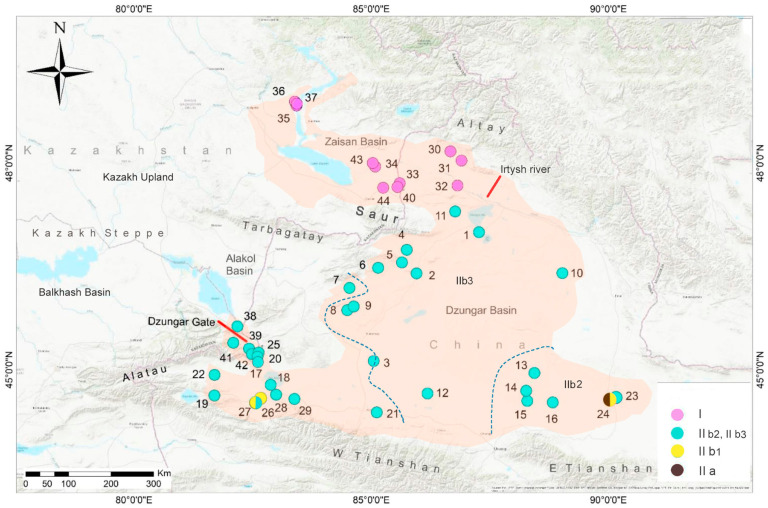
Collection sites for the samples of *P. melanurus* used in this study. Sites are numbered as in Table 1 and Table S1; phylogenetic lineages (clades/Subclades) are highlighted by different colors. Dashed lines represent the soft boundaries isolating the populations of IIb2 (East), IIb3 (Central), and the western group, respectively. The background outlines the current distribution of *P. melanurus* according to Dunayev et al. [34].

One example of such drylands is arid Central Asia. It is well accepted that late Cenozoic progressive aridification in Central Asia is one of the most prominent climate changes in the Northern Hemisphere [12,13,14], resulting in the emergence of diverse arid landscapes, including sandy deserts, and rocky deserts like the Gobi [15]. Meanwhile, the stepwise desertification of Northwest China (NW China) contributed to the emergence of the Guerbantonggut Desert, the second largest sandy desert in Northwest China [16]. This desert is a major component of the Dzungar Basin, which is located in the Northern part of Xinjiang of China. Being an intermontane basin, Dzungar Basin is bounded by Dzungarian Alatau, Tarbagatay, and Saur mountains at the west, Tianshan Mountains at the south, and Altai Mountains at the north (see Figure 1). The formation of the sandy deserts and the Tianshan Mountains has played a crucial role in diversifying the regional environmental conditions [17]. This complex landscape has shaped rich biodiversity and makes the area an ideal system for examining species evolution from a phylogeographic perspective [18,19,20,21]. 

As such, plant phylogeographic studies in arid Northwest China have gained broad prominence [22,23,24]; however, animal phylogeographic studies, particularly those of lizards, are still relatively limited [8,11,21,25]. In fact, lizards are useful for studying the effects of environmental and geological conditions on phylogeographic structure due to their lower dispersal abilities, and susceptibility to climate fluctuations [26,27]. Nevertheless, a general conclusion has not been drawn on how the flora and fauna in Northwest China responded to the Quaternary climatic oscillations [23,28].

*Phrynocephalus melanurus* Eichwald, 1831, also known as the Zaisan toad-headed agama, is one of the members of the *Phrynocephalus guttatus* complex that is highly adapted to sandy and gravel deserts [29]. Its distribution spans from the Zaisan and Alakol (Dzungar Gate vicinity) basins in the Eastern Kazakhstan (E KZ) to the Dzungar Basin in Northwest China [29,30,31]. Due to adaptation to the changing environments, the lizard has become specialized in accommodating the heterogeneous structure of the habitat and forming “substrate races” [32]. This intricate process has given rise to an array of morphological variations that display discernible phenotypic disparities, which have historically led to their classification under diverse taxonomic designations (reviewed by [33] and references therein). The species status of *P. melanurus* has been disputed, and was historically classified as a subspecies of *P. guttatus* [33,34,35]. Recent work suggested that the morphological differences between *P. melanurus* and *P. guttatus* may be corroborated by genetic data, with a high mean sequence divergence of 6.9% in mtDNA *ND2* gene [3], and of 2.48% in mtDNA *COI* gene [34]. Based on limited sampling (eight individuals restricted to two localities), Melville et al. [3] initially found that *P. melanurus* comprises one lineage from the Dzungar Basin (Xinjiang, China) and another from the Zaisan Lake area. Dunayev et al. [34] confirmed the differentiation of *P. melanurus* in Kazakhstan into two lineages inhabiting the Zaisan and Alakol depressions. However, one potential shortcoming of Dunayev et al. [34] lies in the lack of adequate sampling of *P. melanurus* from its whole distribution. There are two passages connecting Dzungar with Kazakh Steppe (Great Dala) through the Dzungar Gate (western) and Irtysh valley (northwestern) (see Figure 1). Thus, agamas could migrate between Dzungar and adjacent Kazakhstan despite partial isolation of this region by mountains.

Therefore, our analyses were motivated by the goal of obtaining a more detailed picture of the genetic structure and to trace the population history of *P. melanurus* across its range. We used a phylogeographic approach complemented with species distribution models (SDMs). Specifically, we aimed to (i) document the phylogeographic structure and the timing of genetic diversification within the Zaisan toad-headed agama; (ii) reconstruct the center of origin and colonization routes; and (iii) explore the relationship between historical demographic changes and past climate fluctuations. Analyses of the mtDNA sequences tell only one part of a potentially more complex story [36,37], yet they provide valuable insights into the evolutionary history of a species, including consequences of habitat changes, impacts from climate fluctuations, divergence, and colonization. To incorporate the data published by Solovyeva et al. [38] and especially the data from the Zaisan Basin [34], we amplified the mitochondrial *COI* gene segment in this study.

## 2. Materials and Methods

### 2.1. Population Sampling

A total of 165 individuals of *P. melanurus* were collected from 36 sites across its whole range from 2008–2019 (Figure 1) including 1 sample from the type locality (Kyzylkum sands (previous name—Bukon sands), of the Kurchum district, Eastern Kazakhstan), which covers the Northern Xinjiang region of China, and the adjacent eastern part of Kazakhstan. Additionally, 10 samples of *P. melanurus* from Kazakhstan (the Zaisan and Alakol basins) were taken from the previous studies [34,38]; 2 outgroup sequences were retrieved from GenBank (https://www.ncbi.nlm.nih.gov) (accessed on 15 March 2021)—*Phrynocephalus alpherakii* (KF691705), *Phrynocephalus guttatus guttatus* (MK461381). Detailed sampling information is listed in Supplementary Materials, Table S1. Animals were euthanized with an overdose of sodium pentobarbital via intraperitoneal injection, and liver tissues were extracted and preserved in 95% ethanol following the animal-use protocols approved by Chengdu Institute of Biology (CIB), Chinese Academy of Sciences. Liver or tail tissue from specimens was fixed with 95% ethanol and stored at −20 °C before DNA extraction. The voucher specimens for all populations are deposited in CIB.

### 2.2. DNA Isolation, PCR Amplification, and Sequencing

Genomic DNA was extracted either from the 95% ethanol-preserved tail or liver tissue samples using the universal high-salt procedures [39]. Amplification of the COI gene fragment was implemented by using the pairs of primer: *PhCOIf* (5′-AATTCAGCCATCTTACCATGTCAAC-3′), *PhCOIr* (5′-TATACTTCTGGGTGGCCAAAGAA-3′), which was designed particularly for this study. The length of amplified sequences was 680 bp. Each PCR reaction contained 25 μL of Taq PCR Master Mix (Omega Bio-Tek), 2 μL each primer (0.4 μM), 1−2 μL genomic DNA (~50 ng), and 19−20 μL double sterilized water for a total reaction volume of 50 μL. The PCR protocol involved an initial denaturation at 94 °C for 4 min, followed by 35 cycles at 94 °C for 30 s, 55 °C for 30 s, and elongation at 72 °C for 53 s, and a final extension at 72 °C for 10 min. The PCR products were assessed using 1% agarose gel electrophoresis, purified, and sequenced for double strands with the PCR primers. All fragments were sequenced with ABI 3730 automated DNA Analyzer at Sangon Biotech (Shanghai, China).

### 2.3. Phylogenetic Reconstruction

All obtained nucleotide sequences were checked and assembled using SeqMan II program included in LASERGENE 7.0 software package (DNAStR Inc., Madision, WI, USA). In total, 177 sequences—which included those from [34,40] (available in GenBank)—were aligned using Clustal X v.1.81 [41] under default parameters. Subsequently, the aligned sequences were translated to amino acids with SeaView v.5.0.1 [42], and no stop codons were detected. Identical sequences were collapsed into a single haplotype using DnaSP v.6.0 [43].

Phylogenetic inference was used to establish the relationships among the observed haplotypes and their associated populations of *P. melanurus*. We used Bayesian inference (BI) and maximum likelihood (ML) approaches to reconstruct phylogenetic relationships among the mitochondrial haplotypes. Bayesian analyses were implemented using MrBayes v.3.2.6 [44] employing partition-specific modeling. The best-fit models of nucleotide substitution for each partition scheme were selected using PartitionFinder v.2.1.1 [45]. Three codon partitions and their corresponding substitution model for the *COI* gene sequences were proposed: first codon—K80 + I; second codon—HKY; third codon—GTR + G. Two simultaneous parallel runs were performed with four heated Markov (using default heating values) chains per run (three heated and one cold) for 10 million generations with sampling frequency of every 1000 generations. Convergence of the runs was assessed by the effective sample sizes (ESS) (≥200) using Tracer v.1.7 [46] and the average standard deviation of split frequencies <0.01. The first 25% of trees were discarded as burn-in and a 50% majority-rule consensus tree was constructed to calculate the posterior probabilities (PPs) of nodes. Partitioned maximum likelihood (ML) analyses were carried out in RAxMLHPC v.8.2.4 [47] with the same partitioning strategy as for BI. The GTR + G model was used for all subsets, and 100 replicate ML inferences were performed for each analysis. Each inference was initiated with a random starting tree and nodal bootstrap support (BS) was assessed with 1000 pseudoreplicates [48].

### 2.4. Divergence Times Estimation

To estimate the divergence time for mitochondrial haplotypes of *P. melanurus* we conducted the Bayesian dating in BEAST v.1.8.4 [49]. Owing to lack of a reliable fossil record and substitution rate of *COI* for *P. melanurus*, we applied as secondary calibration points the estimated mean values of internal nodes of Clades of *P. guttatus-versicolor* species complex (6.5 million years ago, Ma) and *P. guttatus* specie complex (5.0 Ma) from the previous study [40]. Additional sequences were retrieved from GenBank (Supplementary Materials, Table S1) and incorporated with our dataset in order to expand the dataset and re-calculate the divergence age of *P. guttatus* species group. The taxon sets were defined in two groups: *P. guttatus-versicolor* and *P. guttatus* group. The HKY + G site substitution model was used, under the uncorrelated lognormal clock model. The calibration was implemented as a normal prior with standard deviation equal to 1.0; mean values were set as 6.5 Ma and 5.0 Ma, respectively. A birth–death prior was used for the tree. Non-partitioned dataset was used for a single run. Analysis was run for 10 million generations, with random starting tree. Convergence of the four runs was assessed by ESS ≥ 200. The first 10% of generations were discarded as burn-in using LogCombiner, and TreeAnnotator was used to infer the ultrametric tree [49]. We interpreted PP ≥ 0.95 to be strongly supported [50]. Final trees were visualized and edited in FigTree v.1.4.4 (http://tree.bio.ed.ac.uk/software/figtree/) (accessed on 21 April 2021) [51].

### 2.5. Genetic Diversity and Population Genetic Structure Analyses

Haplotypes were extracted using DnaSP v.6.0 [43]. Population genetic diversity was quantified using indices of polymorphic site, number of mutations (m), nucleotide diversity [52], haplotype diversity, number of haplotypes (Nh), and average number of nucleotide differences (k), which were calculated in DnaSP. Uncorrected pairwise sequence divergences (*p*-distances) between and within phylogroups were calculated using MEGA v.X [53]. To infer geographic distribution and relationships of the *P. melanurus* haplotypes, a median–joining haplotype network was constructed using PopART v.1.7 [54].

The grouping of the population was performed via spatial analysis of molecular variance (SAMOVA) using SAMOVA v.2.0 software [55]. The analysis was run for values of *K* ranging from 2 to 8. The number of groups was selected according to *F_CT_* value (the differentiation of groups) using the sum of squared differences between haplotypes, with 100 simulated annealing processes. The configuration of *K* that had one or more single-population groups were excluded due to the group disappearing [56,57]. To estimate genetic variation within populations, among populations within groups and between groups (phyloclades, and as identified by SAMOVA), AMOVA was carried out in the program Arlequin v.3.11 [58], with significance test based on 1000 permutations. Subdivision of two regions represented the Zaisan Basin population and the Dzungar Basin population, which is in accordance with Clade I and Clade II, respectively. Another AMOVA test for four groups, representing four geographic regions North (Clade I), Central (Subclade IIb3), West (group containing sampling sites 3, 17–22, 25, 27–29, 38–39, 41–42), and East (Subclade IIb2), was also conducted.

To test the spatial genetic structure of populations, isolation by distance (IBD) [59] was determined by testing the correlation between geographic distance and pairwise *F_ST_*/(1 − *F_ST_*) using the Mantel test with Mantel test with GenAlEX v.6.5. [60]. The procedure was implemented separately on Clade I and II with 999 number of permutations, under the default parameters. Geographic distances among populations were estimated in R package using package Vegan v.2. (https://CRAN.R-project.org/package=vegan, accessed on 21 April 2021) [61].

### 2.6. Inference of Demographic History

Mismatch distributions of pairwise nucleotide differences were applied to test the sudden demographic expansion for the matrilines of *P. melanurus* using DnaSP, with 10,000 coalescent simulations. The goodness-of-fit between observed and expected distribution was tested by calculating Harpending’s Raggedness index (*Rg*) and the sum of squares deviation (*SSD*). Additionally, three types of neutrality test statistics were applied: Tajima’s *D* [62], Fu’s *Fs* [63], and *R*_2_ statistic [64] calculated in DnaSP as additional assessments of possible population expansion. Significant values of the Tajima’s D and Fu’s *Fs*, as well as *R*_2_ statistics were taken as evidence of population expansion.

Bayesian skyline plots (BSP) [65] were implemented in BEAST v.1.8.4 [49] to estimate the changes in effective population size on an evolutionary time scale. We applied a strict molecular clock with a mean substitution rate (0.012 per site per million years) obtained from the BEAST analysis described above. Owing to the small sample size (three sequences) for the Subclade IIa, BSP analysis was not conducted. We used HKY substitution model with empirical base frequencies, and three partitions into codon positions. Tree parameters were set to randomly generated starting tree and piece-wise constant Skyline model with default number of groups (m = 10), except Subclade IIb1, where we reduced the number of groups to m = 5, due to its relatively small sample size—15 sequences. This method allows the prevention of the over-parameterization of the model that would lead to biased results [66]. The initial 10% of steps are discarded as burn-in. We obtained consistent demographic inferences across three replicates of the analysis visualized by Tracer v.1.7 [46].

### 2.7. Phylogeographic Diffusion in Continuous Space

Following Shi et al. [10], we reconstructed the spatiotemporal history of *P. melanurus* throughout their distribution using the approach of Bayesian phylogeographic diffusion [67] in continuous space implemented in BEAST v.1.10.4 [49]. We analyzed a total of 175 individuals representing 44 sampling localities (Supplementary Materials, Table S1).

We applied a Yule speciation process as a tree prior, Cauchy Relaxed Random Walk (RRW) as a continuous trait model for spatial diffusion, and a strict molecular clock model with a substitution rate of 0.012 per site per million years estimated in this study. Geographic coordinates were provided for all sequences, adding a random jitter to tips with a ±0.50° window size to create unique coordinates for individuals collected at identical sites. Analyses were run for 50 million generations, sampling every 5000 generations. The convergence of the MCMC chains were checked using Tracer v.1.7.1 [46] to ensure adequate mixing and convergence. Finally, the sampled trees were annotated using TreeAnnotator v.1.10.4 and the final tree analysed in SpreaD3 v.1.0.7 [68] to visualize the ancestral area for each lineage.

### 2.8. Past, Present, and Future Distribution Models

To reconstruct the potential species distribution loss-expansion scenarios of the *P. melanurus*, we utilized occurrence data from our field surveys, the resources of Global Biodiversity Information Facility (GBIF: http://www.gbif.org/) (accessed 24 April 2023) [69], and the records and the information of the published literature. The total amount of the occurrence points consisted of 44 localities, where 32 localities were from Northwest China (Xinjiang Uygur Autonomous Region) and 12 localities were from Eastern Kazakhstan (Zaisan basin—8, Alakol basin—4), respectively. The GBIF data were carefully inspected where the distribution sites without coordinates, low precision (decimal < 2), and duplicate coordinates were removed from initial data. To ensure that input occurrence data are spatially not correlated and to reduce sampling bias, all sampling records were rarefied at a spatial distance of 10 km using SDMtoolbox v.1.1c [70] in ArcGIS. After rarefication 38 sampling sites were retained (Supplementary Materials, Table S4) and employed for the modelling.

We downloaded 19 bioclimatic variables for the present (1960–1990), past (last interglacial (LIG; 0.14−0.12 Ma), the last glacial maximum (LGM; 0.026−0.019 Ma), and the late Holocene, Meghalayan period (LH; 0.0042−0.0003 Ma) [71]), and future timelines 2070 (averaged for 2061–2080) at a 2.5 arc—minute resolution (5 km × 5 km) grid from the WorldClim database (http://www.worldclim.org) (accessed on 24 April 2023) [72]. The study area was clipped to 74° E to 97° E and 41° N to 52° N, containing plus a 200 km buffer to the species range. To avoid strong correlation between environmental variables that may cause the overfitting [73], we discarded those variables with Pearson correlation coefficients r > 0.6 based on pairwise comparison of raster files in SDMtoolbox [70], whereas six retained variables (bio1, bio2, bio3, bio8, bio12, and bio15) were used for subsequent analysis. Low-correlated climatic variables obtained for current time were considered for past and future timeline projections employing the model for interdisciplinary research on climate (MIROC6) [74]. The prediction of species distribution in the near future (2070s) was extracted from the WorldClim database represented by three climatic scenarios of greenhouse gas concentration: optimistic (SSP1-2.6), intermediate (SSP2-4.5), and pessimistic (SSP5-8.5) under the Shared Socio-economic Pathways stipulated by the General Circulation Models (GCM) [75,76].

The SDM was executed in MaxEnt v.3.3.3 using a maximum entropy algorithm [77]. Maxent analyses were carried out under the parameters: 70% of the species records were used for training and 30% were used for testing the model with 50 bootstrap replicates. We used the ENMeval package [78] in R to manage model complexity and determine the optimal combination of MaxEnt feature classes and regularization multipliers. The optimal model had a regularization multiplier of 0.1 and a linear/quadratic (LQ) features class (Supplementary Materials, Table S3). The retained parameters were set as the default.

We employed the jack-knife test and utilized the built-in response curves [79] of the MaxEnt software to assess the individual contributions of each environmental variable in our models. Additionally, to identify climate extrapolation across different time periods, we employed the multivariate environmental similarity surfaces (MESS) analysis [80], which is integrated into the MaxEnt software. Since our models were constructed based on present climate variables from the period 1960–1990, results interpretation must be with caution for the areas beyond the current climate range [80]. The MESS analysis provides a value on a scale of −100 to 100, where negative values indicate regions with novel variable values, and a larger absolute negative value signifies a greater deviation from the present conditions. A value of zero suggests variable conditions just before reaching the out-of-range threshold. Positive values indicate a similarity between variables from different time periods and the present, with a positive value close to 100 indicating a closer resemblance to the present conditions [80]. The importance of each variable was assessed based on percent contribution values reported in MaxEnt’s output files. The area under the receiving operator characteristics curve (AUC) was used to evaluate the model reliability of the prediction results, ranging from 0.5 to 1.0, and AUC > 0.7 indicates a fair model. The potentially suitable distribution area of each time period was calculated in ArcGIS based on SDMtoolbox [70].

## 3. Results

### 3.1. Sequence Characteristics

The final alignment of *COI* gene fragment was 630 bp, in which 64 sites were variable across 165 individuals, with 15 singleton variables sites and 49 parsimony-informative sites. No indel (insertion or deletion) was observed in the alignment. The average frequencies of C, T, A, and G were 27.6%, 27.3%, 31.1%, and 14.0%, respectively. The A/T contents (58.4%) were notably higher than the C/G contents (41.3%). A total of 50 haplotypes were identified. Among these, 11 haplotypes were shared by individuals from several sampling sites. Haplotypes 6 and 3 were the most frequent one that comprised in H6—7 sampling sites (1, 2, 3, 5, 6, 10, 11), and in H3—5 sampling sites (20, 25, 27–29), respectively. Five haplotypes were shared by as many as three sampling sites: H2—sites 24, 26, 27; H9—sites 32–34; H13—sites 1, 2, 4; H16—sites 13, 16, 23; H27—sites 17, 22, 25. Four haplotypes were shared by two sampling sites: H24—sites 19, 22; H30—sites 17, 25; H38—sites 6, 11; H49—sites 35, 36. Haplotype diversity (*Hd*) among populations was very high, ranging from 0.182 to 1.000, while nucleotide diversity (*π*) was relatively low, ranging from 0.00029 to 0.01138 (Table 1). Total *Hd* for all populations was 0.950 ± 0.00006, while *π* was 0.01443 ± 0.00075 (Table 2). All new sequences were deposited in GenBank with accession numbers MW856918–MW857082 (Supplementary Materials, Table S1).

### 3.2. Phylogenetic Relationships

**Figure 2:**
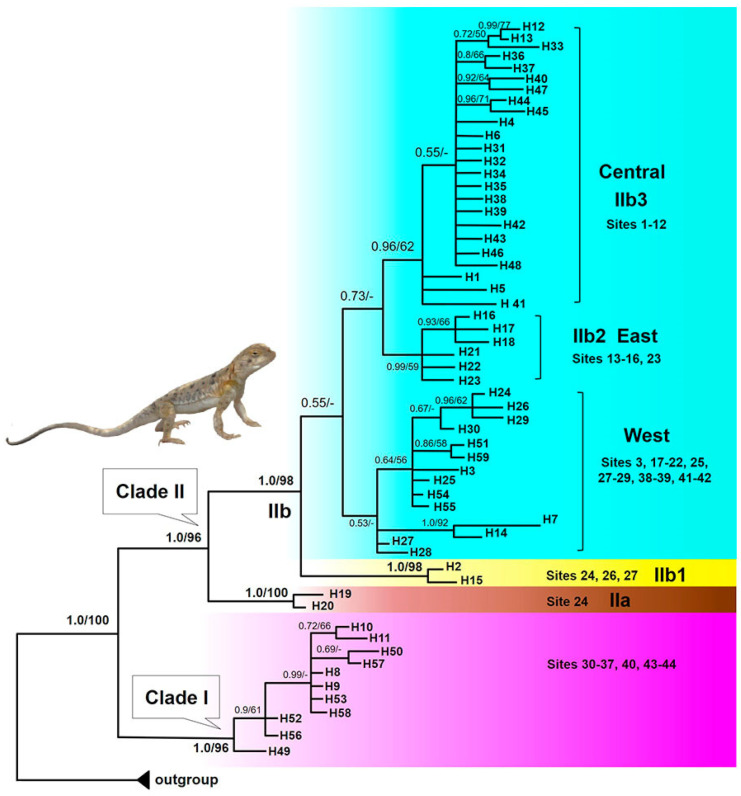
The 50% majority-consensus tree for *P. melanurus* resulting from partitioned Bayesian analysis, associations with less than 0.5 posterior probability being collapsed. Bayesian posterior probabilities and maximum likelihood bootstrap values are shown. Nodal support less than 50% was not shown in the tree. Highly supported Clades/Subclades (>0.95%) are given in bold. Dashes represent nodes with bootstrap support lower than 50% or represent nodes non-existant. Geographic attribution represents Central, West, and East groups of Dzungar Basin populations, respectively. Photo of *P. melanurus* by X.G.

Bayesian inference and ML analyses produced highly congruent topology, with only minor conflicts on recent nodes. Thus, only the BI tree with both PP and BS from ML is presented (Figure 2). Two main Clades were well supported, with high PP and BS values. Clade I (PP = 1.0, BS = 96%) covered the NW part of the Dzungar Basin, comprising the haplotypes of Altay Prefecture in NW China, and the Zaisan Basin in Eastern Kazakhstan. Clade II consisted of two matrilineal lineages, with IIa representing the haplotypes of Qitai (PP = 1.0, BS = 100%) and IIb representing the rest sampling sites. As can be seen from Figure 2, the intra-relationship within IIb was unresolved. IIb1 included the haplotypes of Jinghe (PP = 1.0, BS = 100%), and the West group with low support (PP = 0.53) comprised the haplotypes of western part of the Dzungar Basin and adjacent Dzungar Gate, and Alakol Basin area in Eastern Kazakhstan, while the East Subclade IIb2 represented haplotypes of Fukang (PP = 0.99), with Central Subclade IIb3 of Hoboksar (PP = 0.96). Overall, the genealogical structure was significant, which reflects a strong geographic association of each lineage.

### 3.3. Timeframe of the Diversification

**Figure 3:**
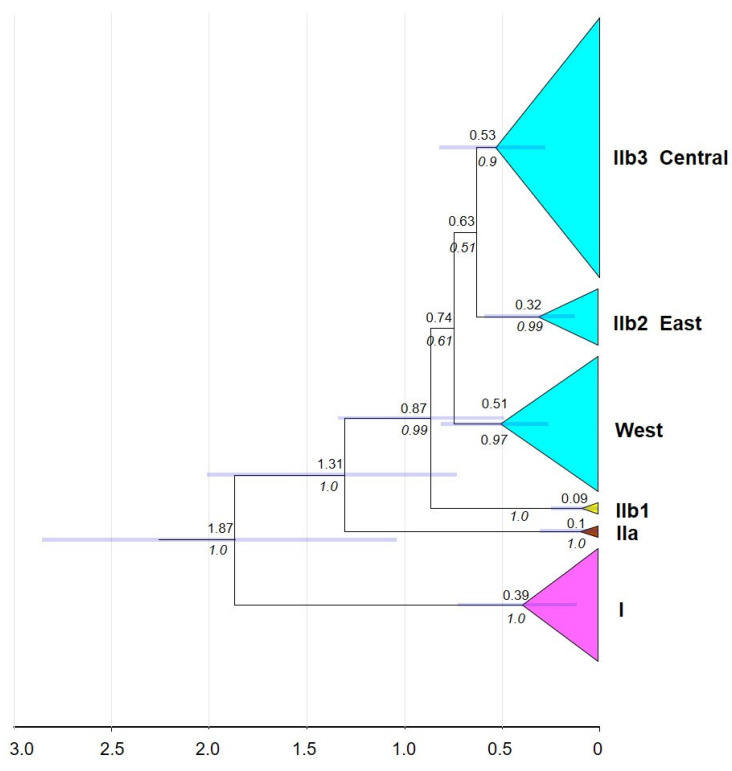
Bayesian divergence times estimation for *P. melanurus* based on the sequences of mtDNA *COI* gene fragment. Median values of divergence (in millions of years ago) are shown above nodes, with blue bars representing 95% highest posterior densities (HPD), and posterior probabilities (in italics) are shown below nodes.

The most recent common ancestor (MRCA) of *P. melanurus* and the closely related outgroup diverged approximately 3.87 Ma (Supplementary Materials, Figure S1). As shown in Figure 3, the MRCA of *P. melanurus* was dated in the early Pleistocene (~1.87 Ma; 95% HPD: 1.04–2.85 Ma). Subsequent divergences within *P. melanurus* occurred at various times throughout the Pleistocene. Within Clade I divergence was estimated to be ~0.39 Ma (95% HPD: 0.14–0.74 Ma). Within Clade II divergence occurred at approximately 1.3 Ma (95% HPD: 0.73–2.01 Ma). Separation of IIb1 from the remaining Subclades took place around 0.87 Ma (95% HPD: 0.48–1.34 Ma), whereas divergence within IIb1 dated at ~0.09 Ma (95% HPD: 0–0.24 Ma), and IIa at ~0.1 Ma (95% HPD: 0–0.3 Ma) in the late Quaternary. Splits within IIb2 and IIb3 occurred at 0.32 Ma and 0.53 Ma, respectively. Discordance of branch lengths and node support was captured for the West group which contains sampling sites (3, 17–22, 25, 27–29, 38–39, 41–42) between Bayesian phylogenetic tree (PP = 0.53; Figure 2) and the timetree (PP = 0.97).

### 3.4. Population Genetic Structure

**Figure 4:**
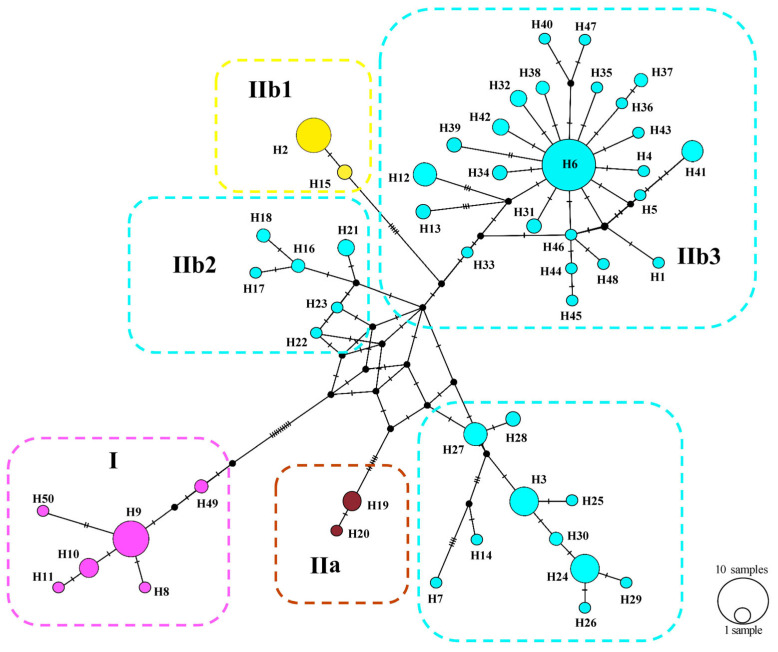
Median–joining networks of mtDNA *COI* haplotypes for *P. melanurus*. Colors correspond to haplotypes of lineages/group in Figure 2. Short bars crossing network branches indicate mutation steps; small dark circles indicate median vectors inferred by PopART software. Circle size corresponds to relative numbers of individuals sharing a particular haplotype.

A median–joining network demonstrated the relationships of the 50 haplotypes (Figure 4). The main feature of the haplotypes’ distribution was the occurrence of an apparent geographic structure, similar to the phylogenetic reconstruction with six structured haplogroups. A star-shaped network in IIb3 suggested that many haplotypes differed from H6 by only one or two mutations, which suggested that IIb3 experienced a population expansion event. Uncorrected genetic distances (*p*-distances) ranged from 1.4–3% between Clades/Subclades (Table 3).

The analysis of spatial genetic structure showed that the *F_CT_* values did not have the highest differentiation among groups when *K* = 5; however, one or more groups contained a single population when *K* ≥ 5. Therefore, we retained the configuration of *K* = 5 with overall *F_CT_* = 0.771 under the Tamura molecular distance model. Group 1 contained populations 1–12; Group 2 incorporated populations 13–16, and 23; Group 3 comprised populations 17–22, 25, and 28–29; Group 4 included populations from 24, 26, and 27; Group 5 contained populations from 30 to 36, which is concordant with Clade I.

The AMOVA analyses were performed on two regions (the Zaisan and Dzungar basins)—Clade I and Clade II, and on four geographic regions (North, Central, West, East). The hierarchical analysis demonstrated that 73.94% or 73.11% of the variation was explained by the variation among two regions or four groups, respectively. Fixation index over all examined data showed significant differences (*p* ≤ 0.001) (Supplementary Materials, Table S5).

The Mantel test for IBD was conducted to estimate the correlation between the genetic distance and geographic distance of *P. melanurus*. If the dispersal of *P. melanurus* is limited by distance, then the genetic and geographic distances should be positively correlated, producing a pattern of isolation by distance. Applying the Mantel test, the weak but significant positive correlation (r = 0.20, *p* = 0.03) between genetic and geographic distance was observed among populations in the Zaisan Basin (Clade I). Meanwhile, a moderate and significant IBD (r = 0.412, *p* = 0.01) was observed among populations in the Dzungar Basin (Clade II) (Supplementary Materials, Figure S2). Overall, the correlation between genetic and geographic distance was confirmed by the positive slope of the first order regression line which is significantly different from zero (R^2^ = 0.6298, *p* = 0.01) for the entire sample (Figure S2).

### 3.5. Historical Demography

Demographic analysis was conducted by applying different approaches to all groups except IIa, due to the small sample size. The neutrality tests resulted in non-significant values of Tajima’s *D* and Fu’s *Fs* for all populations except Subclade IIb3 (Table 4). Notably, Subclade IIb2 is characterized by statistically not significant positive value of *D* and a negative *Fs* statistic that indicates the lack of rare alleles and decrease in population size or balancing selection. Conversely, Subclade IIb3 has observed past population expansion based on large and negative *D* and *Fs* values. Significant small values of *R_2_* statistics were captured for Clade I and Subclade IIb3, which also supported the population growth.

Mismatch distribution analysis of Clade I and Subclade IIb3 produced unimodal curves, suggesting rapid population expansion (Supplementary Materials, Figure S3), which was additionally supported by the non-significant values of the *Rg* and *SSD* indices, as well as by small positive *R*_2_ statistics (Table 4). Meanwhile, Subclades IIb1 and IIb2 with the same modality and non-significant *Rg* and *SSD* values, rejecting population stability. Multimodal curves were observed in Clade II and when all populations were pulled together, which indicates the rejection of population expansion.

**Figure 5:**
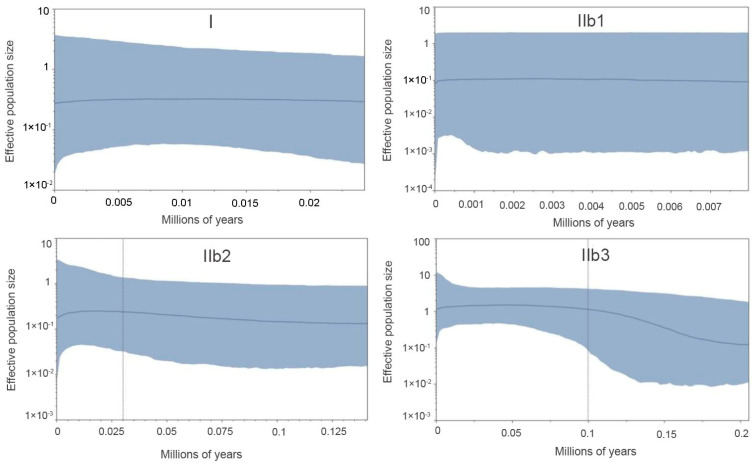
Bayesian skyline plots (BSP) for the Clade/Subclade of *P. melanurus*. The *x*-axis is time in millions of years ago (Ma), and the *y*-axis is on a logarithmic scale and in units of the product of female effective population size (*Nef*) and generation time (t).

The BSP analysis demonstrated the population stability through time in Clade I and Subclades IIb1 and IIb2. Subclade IIb3 detected a past population expansion starting at approximately 0.18 Ma (Figure 5).

### 3.6. The Spatiotemporal Diffusion for P. melanurus

**Figure 6:**
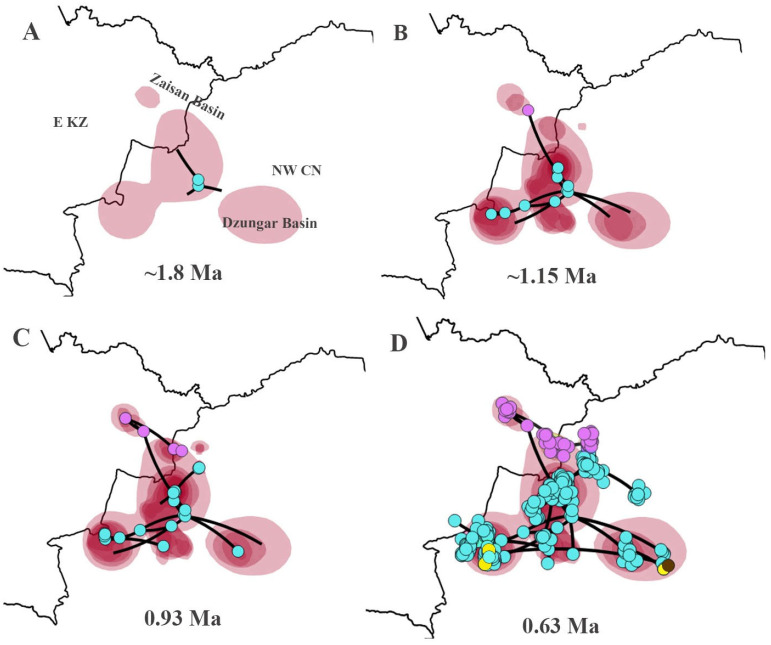
Spatiotemporal diffusion for *P. melanurus* from the potential ancestral area inferred from Bayesian phylogeographic analysis. Four snapshots of colonization events throughout the time are shown: (**A**) the Dzungar Basin origin; (**B**) subsequent multiple spreading northward to E KZ (Zaisan Basin), southward to Karamay region, and southeast to Qitai-Fukang, and southwest through Jinghe and Ebinur reaching Dzungar Gate area; (**C**) the west to east spreading from Zaisan Basin in E KZ to Altay Prefecture in NW CN, and from Bortala Autonomous County to Kuytun area; (**D**) the full event of dispersal with accession to Alakol Basin in E KZ. Colored polygons represent the 80% HPD intervals which indicate the uncertainty of phylogeographic estimates for the nodes. Colored circles represent the samples of maternal lineages according to Figure 2.

For *P. melanurus*, the ancestral area was estimated as the territory of current Hoboksar-Mongol Autonomous County which is in Tacheng prefecture of Xinjiang of Northwest China. The initial colonization event started approximately 1.8 Ma (Figure 6A). The subsequent colonization route followed multiple directions and at 1.15 Ma the population reached Zaisan Basin, Karamay region, westward through Ebinur, Jinghe reached the Dzungar Gate territory, and eastward Fukang-Qitai region (Figure 6B). At ~0.93 Ma local spreading was inferred throughout the main directions and reached the Ulungur territory, Kuytun, and Jimsar regions (Figure 6C). The final dispersal was detected throughout the vast territory of Zaisan and Dzungar basins, reaching the Alakol Basin in E KZ occurred around 0.63 Ma (Figure 6D).

### 3.7. Potential Species Distribution Modeling

**Figure 7:**
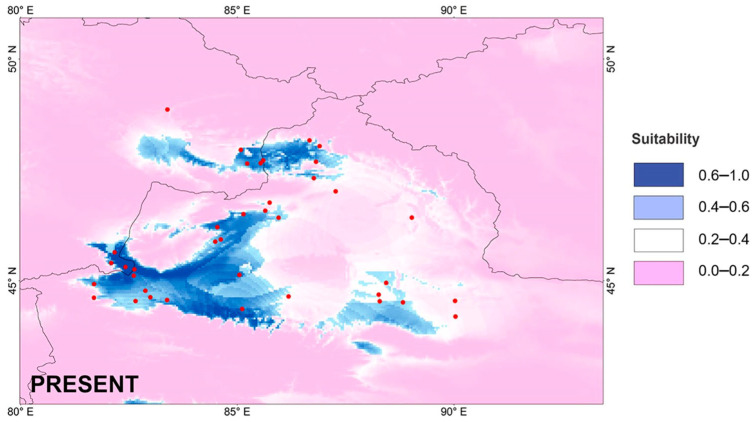
Species distribution modeling for *P. melanurus* at the present time. The range of habitat suitability index indicated as pink (0–0.2)—not suitable; as white (0.2–0.4)—low suitability; as light blue (0.4–0.6)—moderate suitability; (0.6–1.0) with dark blue—highly suitable. Red dots indicate the locality of occurrence data.

**Figure 8:**
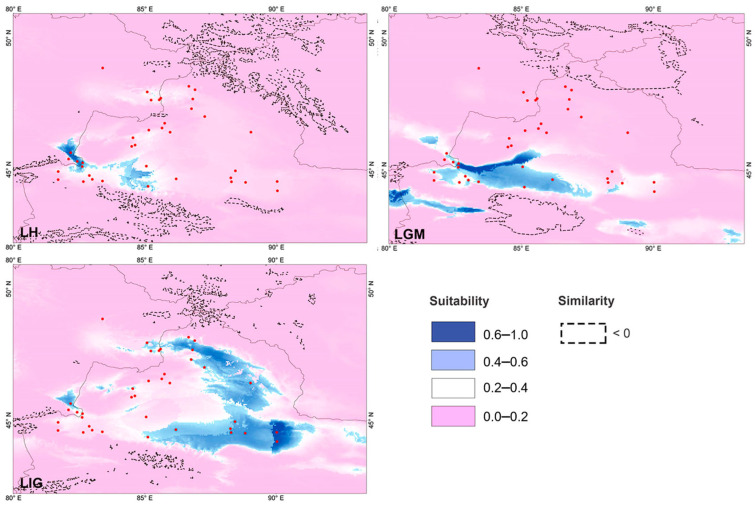
Potentially suitable distribution area in three different periods for *P. melanurus*. LH—late Holocene; LGM—last glacial maximum; LIG—last interglacial period. The habitat suitability index ranged from 0–1; the larger the number, the higher the adaptability of the habitat, and the more suitable for the survival of *P. melanurus*. Negative MESS values shown as similarity <0 by dashed line, demonstrating areas without current equivalents of climatic conditions. Red dots indicate the locality of occurrence data.

The reconstructed potential distributions of *P. melanurus* for past and present-day projections are presented in Figure 7 and Figure 8. The simulation results demonstrated good credibility, as indicated by the AUC values of 0.906 ± 0.019 and 0.878 ± 0.031 for the training and testing datasets, respectively. The jack-knife analysis of regularized training gained from retained environmental variables that highly contributed to the distribution model is shown in Figure S4. Highly contributed climatic variables to the current environment were annual temperature (Bio1)—25.8%; annual precipitation (Bio12)—26.2%; temperature of wettest quarter (Bio8)—18.3%; mean diurnal range of temperature (Bio2)—12% (Supplementary Materials, Table S2). This indicated that temperature and humidity played an important role in the potential geographic distribution pattern of *P. melanurus*. The response curves between the environmental variables and the prediction changes of the occurrence are shown in Figure S5. There was a nonlinear relationship between the probability of occurrence and Bio1. The probability of occurrence peaked when bio1 was 75 °F. The response curve for occurrence also showed a clear nonlinear relationship between the probability of occurrence and Bio3 and Bio12 (Supplementary Materials, Figure S5). The probability of occurrence decreased substantially when Bio2 ranged from 105 °F to 125 °F, with Bio12 ranging from 100 mm to 600 mm. The examination of the response curve profiles for these variables indicates that *P. melanurus* occurs in temperate areas with low levels of precipitation.

The projected LIG scenario showed the broad area of the potentially suitable habitat for *P. melanurus*, concentrated mostly on the eastern margin of its distribution, connecting with the Zaisan Basin through the northern part Dzungar Basin. Modelled LGM climate reconstruction showed the shift of potentially suitable area to southwestern part, which apparently expanded toward Alakol Basin through Dzungar Gates. Late Holocene period simulation was characterized by drastic habitat diminishing, particularly, massive contraction occurring at the northern and southeastern periphery of Northern Xinjiang. Present-day scenario demonstrated a recovery of the northern and eastern habitat corridor approximately as it was in the LIG (Figure 7 and Figure 8); the highly suitable habitat for *P. melanurus* is concentrated in the north, west, and south parts of its range; this is consistent with all known up-to-date occurrence records.

All predicted future projections displayed the broad range habitat reduction in the territory of north, east, and south regions of NW China. Also, the SW area habitat loss (the Dzungar Gate, Alatau) is important to observe. SSP2-8.5 scenario was demonstrated to be slightly different from the SSP1-2.6 scenario, where we observed moderate habitat discontinuity between the W and SW sectors. However, the SSP5-4.5 scenario showed the irreversible loss of habitat throughout the whole range, preserving only the southwestern margin at slopes of the Western Tianshan Mountains.

Projection uncertainty and areas with non-analog climates were assessed using MESS as a quantitative measure. Overall, the MESS analyses showed that climatic conditions were analogous between the present conditions and the different scenarios, except LGM and future periods (Figure 8; Supplementary Materials, Figure S6).

## 4. Discussion

The present study generates the substantive mtDNA data of *P. melanurus* covering its whole distribution, and unravels the spatial genetic structure, demographic history, and divergence dates. Furthermore, the work provides the primary evidence for the species ancestral center and subsequent colonization trajectories emphasized by historical changes of the populations during the Pleistocene climatic oscillations. However, the results should be interpreted with caution, since this study relies on the genetic structure found in a single locus (mtDNA) topology rather in multiple independent evolving loci, which might unravel a more precise evolutionary history of the species by taking into account potential gene tree discordances. Further genetic information, using fast-evolving markers (i.e., microsatellites) or large single nucleotide polymorphism datasets, are needed to better understand the evolutionary history of this species.

### 4.1. Phylogeographic Pattern and Diversification History

By utilizing mtDNA *COI* gene sequences in phylogenetic analysis, we revealed five groups within the two main Clades of *P. melanurus*, where most branches are highly supported (Figure 2). Clade I represents the Zaisan Basin lineage spanning from E KZ to the Altay Prefecture of N Xinjiang in China, while Clade II embodies the lineages of Dzungar Basin (Figure 1). Genetic distances between the Zaisan lineage and the lineages of Dzungar Basin ranged from 2.5 to 3%, which indicates a subspecies status due to an ongoing lineage sorting process and/or gene flow. Similar findings were captured by Solovyova et al. [38] and Dunayev et al. [34], where the Zaisan lineage diverged 2.6% and 2.48% from that of the Dzungar Gate, respectively. Later Solovyova et al. [40] contended the recognition of *P. melanurus* from the Zaisan and Dzungar basins as a distinct species based on *p*-distances of *COI* gene, morphology and geographic distributions. Macey et al. [81] based on *ND1*-*COI* region of mtDNA evaluated the genetic distances between *P. melanurus* (mentioned in that study as *P. salenskyi* populations 1 and 2) from Dzungar Basin and Zaisan Basin populations—2.21% [81].

The population from Northwest China was examined by Wang and Fu [82] and Melville et al. [3], based on the analysis of *ND2* gene sequences. Their results demonstrated that the population of Northwest China is sister to E KZ population, with 3.1–4.0% uncorrected sequence divergence between them.

SAMOVA divided all sampled populations into five groups with certain geographic distribution. Analysis of molecular variance (AMOVA) also suggested that the genetic variation in *P. melanurus* was high among the groups (Supplementary Materials, Table S4). Haplotype 6 is dominant in the population of the northern Dzungar Basin and indicates a potential ancestral haplotype. The haplotype 3 is frequent for the group of IIb1 Subclade, while haplotype 2 is shared between IIa and IIb1, which might have resulted from the secondary contact during last interglacial cycles (Figure 4). All these facts demonstrate that there is a distinct genetic differentiation among the geographic groups. Similar population structures were documented in previous studies of xerophyte plants [83,84].

The haplotype and nucleotide diversity indices of the Dzungar Basin population (Table 1) were detected to be significantly higher than those of the Zaisan Basin. This suggests that *P. melanurus* experienced an expansion and recent stepwise dispersal, which is also supported by the haplotype network analysis revealing a star-shaped topology of IIb3. Similar findings were reported for the desert scorpion *Mesobutbus mongolicus* [10], agamid lizards *Phrynocephalus* spp. [3,11], and the rapid racerunner *Eremias velox* [8].

The estimated divergence time from the MRCA of *P. melanurus* occurred approximately 1.87 Ma (Figure 3), which falls into the early Pleistocene, the phase of intense uplift of Himalayas and Tianshan Mountains [85,86]. This age is in accordance with that of Macey et al. [81], albeit slightly older than the previous studies of Solovyova et al. [40] and Dunayev et al. [34], which might be due to the limited sampling size of *P. melanurus* analyzed by the mentioned authors. The Dzungar Basin lineage initiated a divergence at ~1.3 Ma, with subsequent intraspecific differentiation at the Middle and Late Pleistocene epochs (Figure 3). Notably, Pleistocene climatic transitions placed a unique arid environment in Northwest China [87], which may have shaped the genetic diversity of vertebrate populations, particularly an isolation of desert-dwelling species [88,89].

### 4.2. Origin and Colonization

The origin of the *P. melanurus* has been debated for decades. Previously, researchers hypothesized that an ancestral form of the *P. guttatus* group originated in the eastern part of the Kazakh Upland and later spread to the Zaisan, Balkhash, and Ili basins, eventually settling in western Kazakhstan [32]. Melville et al. [3] assumed that the Irtysh valley could serve as a dispersal route for *P. melanurus* populations, while Golubev [29] proposed a hypothesis that *P. melanurus* may have penetrated into Kazakhstan from China through the Dzungar Gate in the Middle Pleistocene. However, our study highlights that the ancestral form of *P. melanurus* colonized the Zaisan Basin from Chinese Dzungar. Bayesian phylogeographic diffusion analysis showed that the dispersal of *P. melanurus* populations from its projected ancestral area occurred in the middle Pleistocene epoch, which was conditioned by the mid-Pleistocene climatic transition (Figure 6A,B). The subsequent expansions of deserts in the Dzungar Basin (0.65 Ma and 0.5 Ma) [16] uncovered the vast territories for the settlement of *P. melanurus* and promoted the spatial diffusion of the population in the Dzungar Basin at 0.63 Ma (Figure 6D).

Subsequently, following the mountain and foothill trails of Saur-Tarbagatay and Alatau mountains, populations reached Ebi-Nur Lake (IIb1) and spread eastward to the modern settlement of Qitai (IIa). During that period, the activity of shallow rivers and temporary streams along the northern slopes of the Dzungar Alatau could have contributed to the formation of sandy–pebble desert landscapes that eventually developed into a relatively integral piedmont plume [90,91]. This plume may have also served as a pathway for lizards from Ebi-Nur Lake to penetrate the Alakol Basin via the Dzungar Gate, which stands with the Golubev’s assumption [29].

### 4.3. Lineage-Specific Response to Quaternary Climatic Oscillations

We suggest that the historical climate has greatly influenced the population dynamics of *P. melanurus* in Northwest China. Demographic analysis of the lineages examined in this study reveal that the population growth was captured in the lineage IIb3, matching glacial expansion model [26,92]. For Subclade IIb3, a star-like haplotype network, significant values of neutrality statistics and unimodal mismatch distribution evidenced the population expansion event (Figure 4 and Figure S3). Moreover, BSP analysis detected the signal of the past population growth started at approximately 0.18 Ma and lasted during the LIG in IIb3 (Figure 5). SDM modelling of the LIG period demonstrated the suitable habitat expansion at the eastern margins of the area, which supports the possibility of the population expansion in the past (Figure 7). Clade I and Subclade IIb2 kept the constant population over the time, which also shows the stability of the distribution area during the LGM.

The development of aridification in Northwestern China in the late Pleistocene resulted in the enlargement of deserts in the LGM [93], which may in some cases have had the effect of forming broader suitable habitats (edges of deserts and arid piedmont) for expansion of the arid-adapted species. As expected, LGM climatic transition shifted the habitat of *P. melanurus* by reinforcing them to shelter along the mountain slopes in southwestern range, expanding through the Dzungar Gate into Kazakhstan (Figure 8). Suitable habitat expansions during the LGM were noticed in the other representatives of herpetofauna in arid Central Asia, such as the Turpan racerunner (*Eremias roborowskii*) [8] and sunwatcher toad-headed agama (*Phrynocephalus helioscopus*) [11]. In the late Holocene, the suitable habitat contracted due to the warm and humid episodes that have promoted the development of the mesic biota [94].

It is widely accepted that glaciation in the Pleistocene generally forced species into refugia [95,96,97,98]. Refugia may be generally predicted as areas possessing high levels of genetic diversity, and may have a distinct characteristic, such as a presence of ancestral and unique haplotypes that have disappeared from other populations [96]. In our study, the high level of genetic diversity in populations of IIb3, IIa, and IIb1 is detected. Additionally, the potential ancestral haplotype (haplotype 6) comes from the Hoboksar region, which coincides with the projected ancestral area inferred from Bayesian phylogeographic analysis. All these facts indicate that the Hoboksar region may serve as a conditional refugium where the population survived during the interglacial–glacial periods. Nevertheless, it should be noted that Eastern Tianshan Mountains did not advance the glaciers/permafrost below 2400 m a.s.l. during the LGM due to the extra continental climate [85]. As such, multiple glacial refugia may have existed along the Saur-Tarbagatay Mountain system in the west, Alatau and Western Tianshan in the southwest, and Eastern Tianshan (Bogda Mountain) in the southeast.

Future distribution modelling for the 2070 (Supplementary Materials, Figure S6) proposes the scenario of drastic habitat contraction in NW China, as a response to the extensive aridification and probable urbanization growth. Despite on-mass aridification of the land masses on a global scale, arid-adapted species will face biological limitations or physical barriers that restrict their spatial distribution into suitable habitats [99]. Species incapable of migrating are bound to remain in their current habitats and either adapt to new conditions or face extinction. Similarly, for the *P. melanurus* populations in future, climate change can cause reduction in the population size, or even extirpation, and this matter may require the future conservation effort.

## 5. Conclusions

To the best of our knowledge, this work represents the first range-wide phylogeography of *P. melanurus* by integrating mtDNA and species distribution modeling. Our analyses demonstrate the effects of past climatic changes on the intraspecific divergence of *P. melanurus*. The combination of population genetics and SDMs also provides new insights to predict the impact of future climatic changes on population dynamics. Our results reveal that the population of *P. melanurus* is geographically structured into two main Clades: the Zaisan lineage and the Dzungar lineage. The latter is further sub-structured into several groups. Genetic distances among these lineages demonstrate their relatedness, and thus preclude recognition of as distinct species, due to ongoing diversification processes and incomplete lineage sorting. Furthermore, our results suggest that the ancestral form of *P. melanurus* migrated from the northwest of the Dzungar Basin during the middle Pleistocene, and subsequently spread throughout Zaisan and Dzungar basins, with some populations accessing the Alakol Basin in Kazakhstan via the Dzungar Gate. Overall, taking into account the fact that mtDNA is highly variable in natural populations due to its elevated mutation rate, and can generate a signal about population history over short time frames, this work improves our understanding of the phylogeography of *P. melanurus*, providing further insights into the evolutionary processes that occurred in Northwest China.

## Figures and Tables

**Table 1:** Sampling, sample size, haplotype, and nucleotide diversity for 36 populations for *P. melanurus*. Abbreviations: KZ—Kazakhstan; CN—China; N—number of samples; X—longitude; Y—latitude; Hd—haplotype diversity; π—nucleotide diversity; SD—standard deviation.

Site Number	Locality	N	X	Y	Genetic Diversity		
					Haplotypes	Hd ± SD	π ± SD
1	Ulungur, CN	5	87.26	47.01	H6, H12–13	0.700 ± 0.218	0.00254 ± 0.00079
2	Karamay 1, CN	9	85.95	46.41	H6, H13, H32–35	0.889 ± 0.091	0.00300 ± 0.00073
3	Karamay 2, CN	4	85.05	45.13	H6–7	0.500 ± 0.07031	0.00873 ± 0.00463
4	Hoboksar 1, CN	11	85.74	46.76	H13, H42	0.509 ± 0.101	0.00485 ± 0.00096
5	Hoboksar 2, CN	6	85.64	46.57	H6, H36–37	0.733 ± 0.155	0.00180 ± 0.00039
6	Hoboksar 3, CN	10	85.14	46.50	H6, H38–40, H48	0.844 ± 0.080	0.00317 ± 0.00052
7	Emin, CN	1	84.53	46.20	H7	–	–
8	Toli 1, CN	3	84.49	45.88	H4–5	0.667 ± 0.314	0.00529 ± 0.00249
9	Toli 2, CN	2	84.62	45.93	H4	0.000 ± 0.000	0.00000 ± 0.00000
10	Fuyun, CN	6	89.02	46.42	H6	0.000 ± 0.000	0.00000 ± 0.00000
11	Jeminay, CN	13	86.76	47.31	H6, H39. H43–47, H49	0.808 ± 0.113	0.00265 ± 0.00061
12	Shihezi, CN	1	86.18	44.64	H1	–	–
13	Fukang 1, CN	4	88.43	44.95	H16–18	0.833 ± 0.222	0.00185 ± 0.00058
14	Fukang 2, CN	3	88.28	44.53	H17, H21	0.667 ± 0.314	0.00317 ± 0.00150
15	Fukang 3, CN	2	88.26	44.68	H21	–	–
16	Jimsar, CN	4	88.82	44.51	H16, H22–23	0.833 ± 0.222	0.00265 ± 0.00071
17	Ebinur 1, CN	2	82.61	45.11	H28, H31	1.000 ± 0.500	0.00476 ± 0.00238
18	Ebinur 2, CN	1	82.87	44.77	H25	–	–
19	Bortala 1, CN	7	81.69	44.61	H24, H26	0.286 ± 0.196	0.00045 ± 0.00031
20	Bortala 2, CN	1	82.60	45.19	H3	–	–
21	Kuytun, CN	1	85.11	44.36	H14	–	–
22	Bole, CN	11	81.69	44.92	H24, H27–30	0.709 ± 0.099	0.00404 ± 0.00056
23	Qitai 1, CN	1	90.03	44.19	H16	–	–
24	Qitai 2, CN	4	90.02	44.54	H2, H19–20	0.833 ± 0.222	0.01138 ± 0.00554
25	Alashankou, CN	9	82.62	45.25	H3, H28, H31	0.556 ± 0.165	0.00132 ± 0.00055
26	Jinghe 1, CN	11	82.65	44.54	H2, H15	0.182 ± 0.144	0.00029 ± 0.00023
27	Jinghe 2, CN	4	82.58	44.54	H2, H3	0.500 ± 0.265	0.00635 ± 0.00337
28	Jinghe 3, CN	1	82.99	44.62	H3	–	–
29	Jinghe 4, CN	1	83.37	44.56	H3	–	–
30	Bolade, CN	1	86.66	48.16	H11	–	–
31	Buerjin 1, CN	5	86.90	48.03	H10	0.000 ± 0.000	0.00000 ± 0.00000
32	Buerjin 2, CN	9	86.81	47.69	H9	0.000 ± 0.000	0.00000 ± 0.00000
33	Zaisan, KZ	4	85.59	47.71	H8, H9	0.667 ± 0.204	0.00106 ± 0.00032
34	Kurchum, KZ	5	85.08	47.94	H9, H50	0.400 ± 0.237	0.00127 ± 0.00075
35	Kokpekty 1, KZ	1	83.38	48.85	H50	–	–
36	Kokpekty 2, KZ	2	83.42	48.80	H50	0.000 ± 0.000	0.00000 ± 0.00000

**Table 2:** Molecular diversity indices of *P. melanurus* lineages. N—number of individuals; Nh –number of haplotypes; S—number of polymorphic sites; m—number of mutations; k—average number of nucleotide differences; Hd—haplotype diversity; *π*—nucleotide diversity.

Clade/Subclade	N	Nh	S	m	K	Hd ± SD	*π* ± SD
I	27	6	7	7	1.13390	0.661 ± 0.086	0.00180 ± 0.00038
II	138	44	57	60	5.95261	0.942 ± 0.010	0.00945 ± 0.00048
IIa	3	2	1	1	0.66667	0.667 ± 0.314	0.00106 ± 0.00050
IIb1	15	2	1	1	0.13333	0.133 ± 0.112	0.00021 ± 0.00018
IIb2	14	6	6	6	1.89011	0.857 ± 0.056	0.00300 ± 0.00029
IIb3	70	24	29	31	2.46253	0.863 ± 0.034	0.00391 ± 0.00040
All	165	50	64	69	9.08914	0.950 ± 0.00006	0.01443 ± 0.00075

**Table 3:** Uncorrected *p*-distances between groups of *P. melanurus* are shown below the diagonal. Standard error estimate(s) are shown above the diagonal and were obtained by a bootstrap procedure (1000 replicates).

Clade/Subclade	I	IIa	IIb1	IIb2	IIb3
I		0.00550	0.00626	0.00570	0.00585
IIa	0.02534		0.00562	0.00519	0.00517
IIb1	0.03017	0.02180		0.00418	0.00432
IIb2	0.02699	0.02033	0.01463		0.00264
IIb3	0.02874	0.02165	0.01539	0.01095	

**Table 4:** Neutrality tests and mismatch distribution analyses of *P. melanurus*.

Clade/Subclade	Tajima’s *D*	Fu’s *Fs*	*R*_2_	*Rg*	*SSD*
I	−1.13891	−1.418	0.0881 **	0.035	0.001
II	−1.32068	−19.847	0.0506	0.033	0.002
IIb1	−1.15945	−0.649	0.2494	0.427	0.012
IIb2	0.00646	−1.120	0.1459	0.188	0.043 ***
IIb3	−1.96737 *	−15.870 *	0.0397 **	0.014	0.001
All	−0.77779	−13.991	0.0696	0.006	0.010

*SSD*—sum of squared distribution; *Rg*—Harpending’s raggedness index. * *p* < 0.05; ** *p* < 0.01; *** *p* < 0.001.

## Data Availability

All novel sequences obtained in this study were deposited in GenBank under accession numbers MW856918–MW857082.

## Supplementary Materials

The following supporting information can be downloaded at: https://www.mdpi.com/article/10.3390/ani14020209/s1, Figure S1: Bayesian divergence times estimation of *P. melanurus* and *P. guttatus* species complex and *P. versicolor* species complex employing calibration points approach. The estimated divergence times are shown in red for the major nodes in millions of years, along with Bayesian posterior probabilities in italics. Node bars indicate 95% HPD of estimated divergence times. The nodes with red dots are two calibration points. Figure S2: Correlation analysis between the pairwise *F*_ST_/(1 − *F*_ST_) values and the geographic distance based on mtDNA *COI* sequences; Figure S3: Mismatch distribution plot for Clade/Subclade of *P. melanurus*; Figure S4: The Jack-knife analysis of regularized training gain from retained environmental variables that highly contributed to the distribution model; Figure S5: Average response curve profiles for the used variables in SDM for *P. melanurus*. °F, Fahrenheit temperature degree; mm, millimeters. Figure S6: Potentially suitable distribution area for *P. melanurus* in 2070 (2061–2080) under three different gas emission models, Shared Socio-economic Pathway SSP1-2.6; SSP2-4.5; SSP5-8.5. Red dots indicate the locality of occurrence data. Table S1: List of analyzed specimens, geographic origin, Clade assignment and GenBank accession number; Table S2: Environmental variables highly contributed to the SDM for *P. melanurus* with Pearson correlation coefficients *r* ≥ 0.6; Table S3: Maxent model parameterization ranking obtained from R package ENMeval. Table S4: Sampling sites retained after rarefication for species distribution modeling; Table S5: Hierarchical analysis of AMOVA for testing the genetic subdivision of populations for *P. melanurus* using *COI* sequences. Statistical significance at *p* ≤ 0.001.
