# Supplementary material for: Genetic Structure and Population History of the Zaisan Toad-Headed Agama (Phrynocephalus melanurus) Inferred from Mitochondrial DNA
Source: Animals (Basel). 2024 Jan 8;14(2):209. doi: 10.3390/ani14020209 (PMC10812424; doi:10.3390/ani14020209)
Supplement: Supplementary file 1 [file animals-14-00209-s001.zip › animals-2689477-supplementary.pdf]

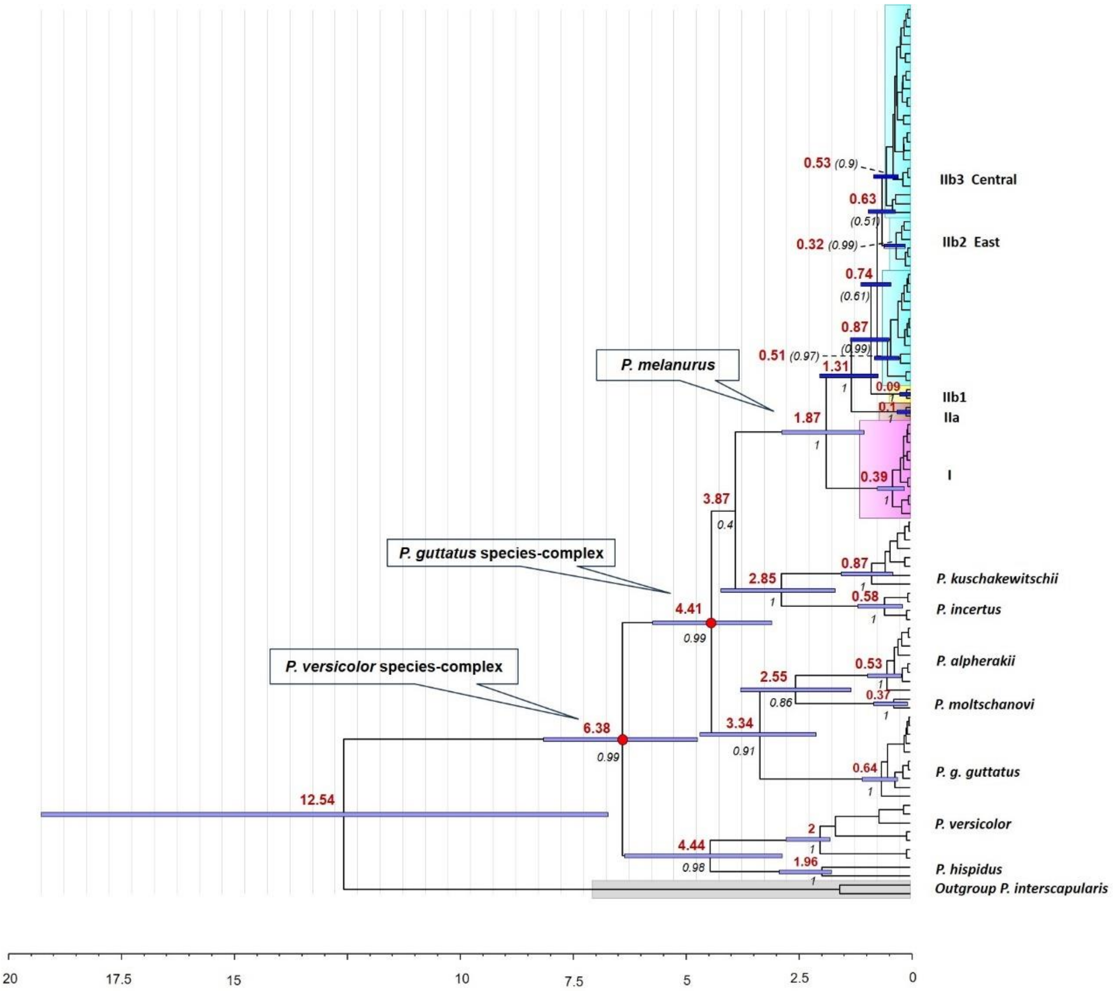

Figure S1. Bayesian divergence times estimation of *P. melanurus* and *P. guttatus* species complex and *P. versicolor* species complex employing calibration points approach. The estimated divergence times are shown in red for the major nodes in millions of years, along with Bayesian posterior probabilities in italics. Node bars indicate 95% HPD of estimated divergence times. The nodes with red dots are two calibration points.

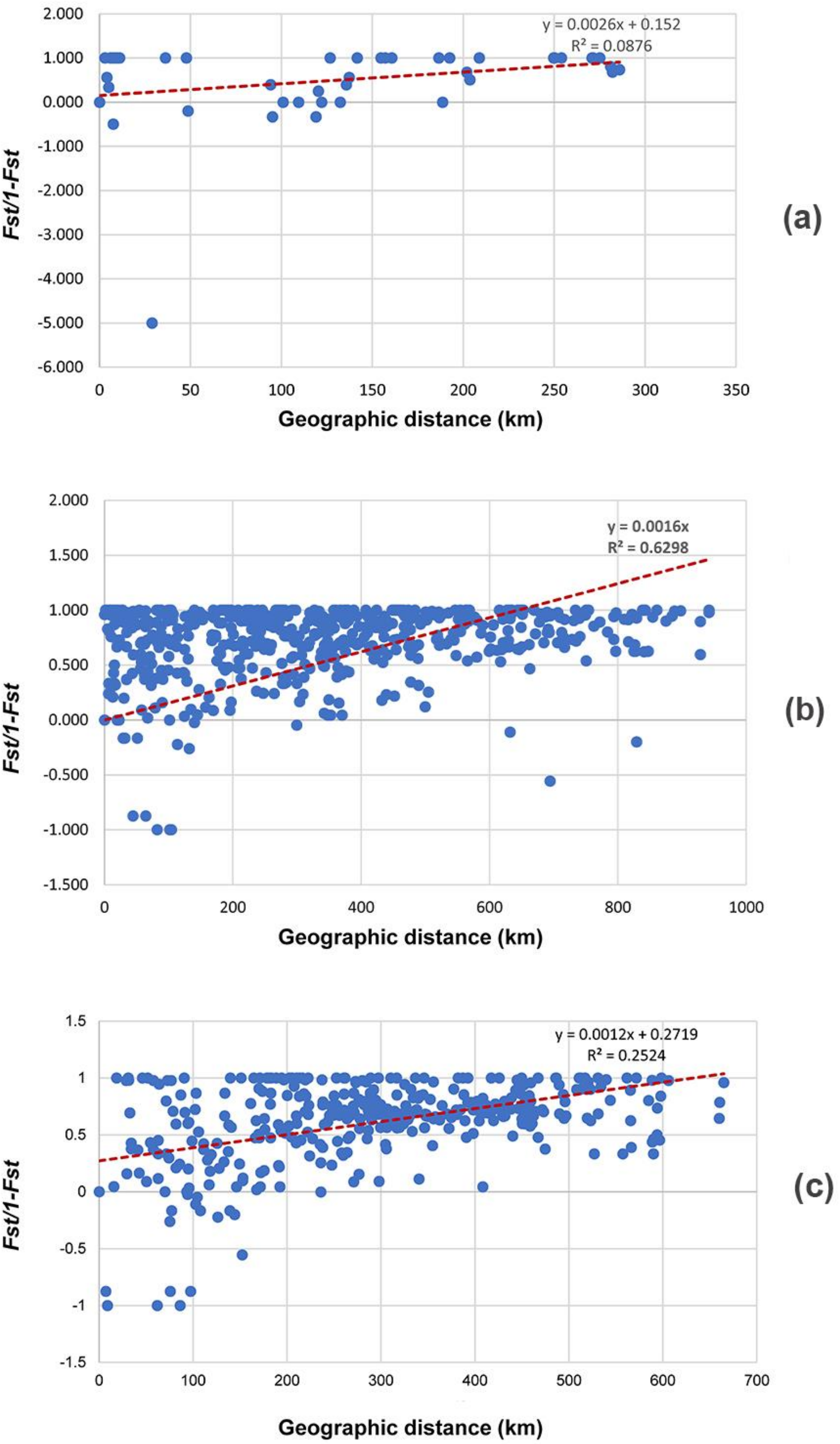

Figure S2. Correlation analysis between the pairwise $F_{ST}/(1–F_{ST})$ values and the geographic distance based on mtDNA *COI* sequences: for (**a**) Clade I, Zaisan populations; (**b**) Clade II, Dzungar populations; (**c**) all data.

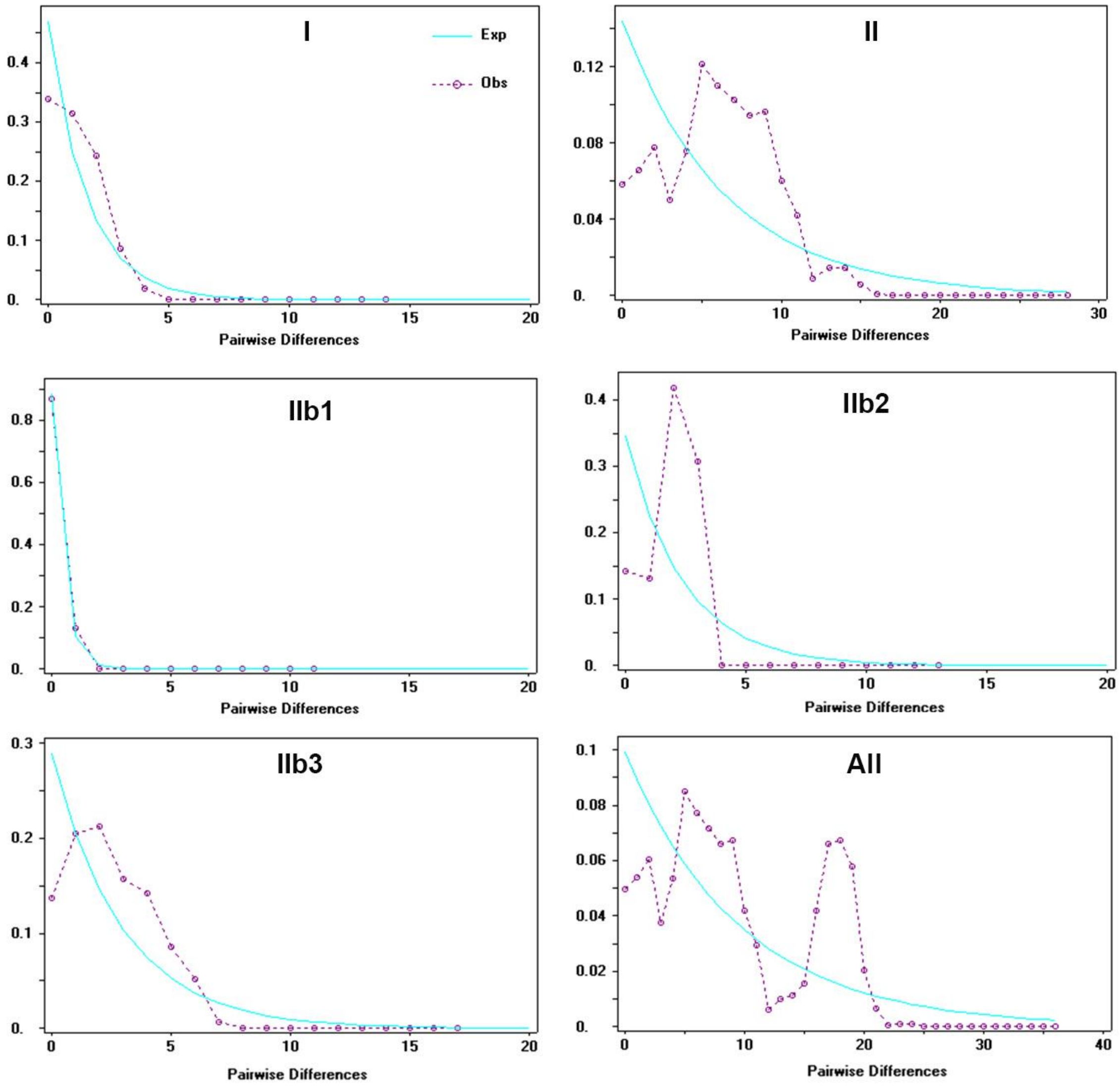

Figure S3. Mismatch distribution plot for clade/subclade of *P. melanurus*. The purple dotted lines are the observed frequencies of pairwise divergences among sequences, while the turquoise solid lines refer to the expected values under a population growth scenario.

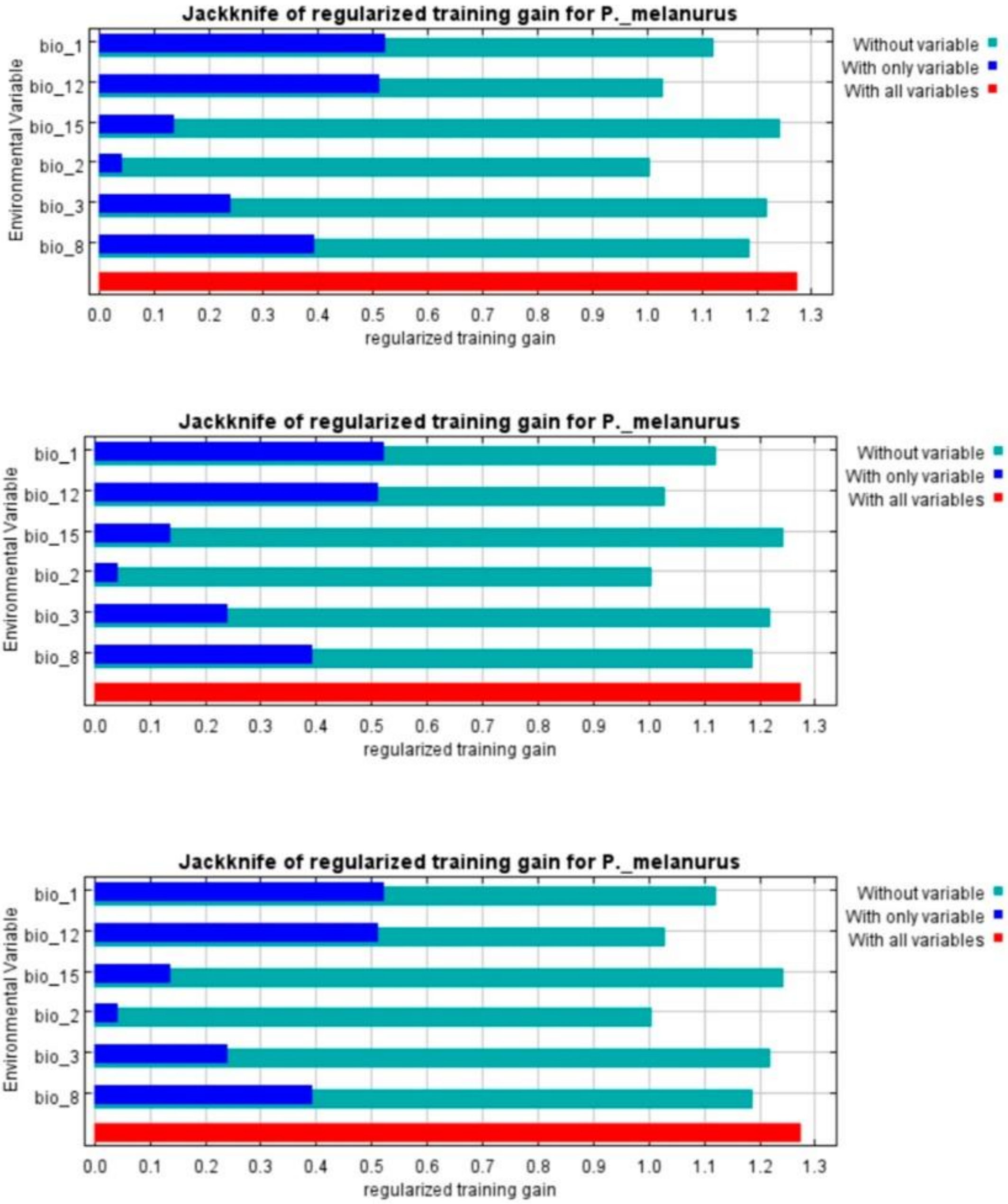

Figure S4. The Jackknife analysis of regularized training gain from retained environmental variables that highly contributed to the distribution model.

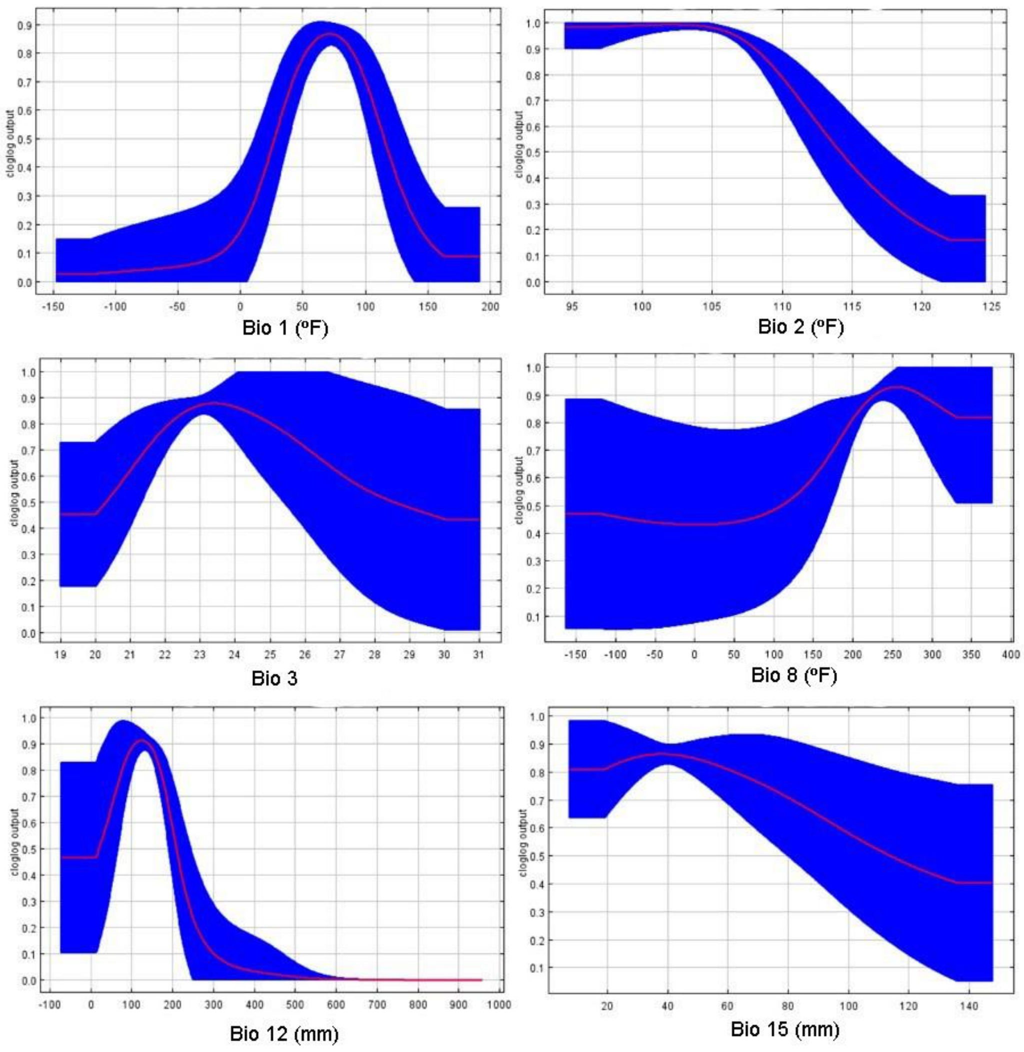

Figure S5. Average response curve profiles for the used variables in SDM for *P. melanurus*. ℉, Fahrenheit temperature degree; mm, millimeters.

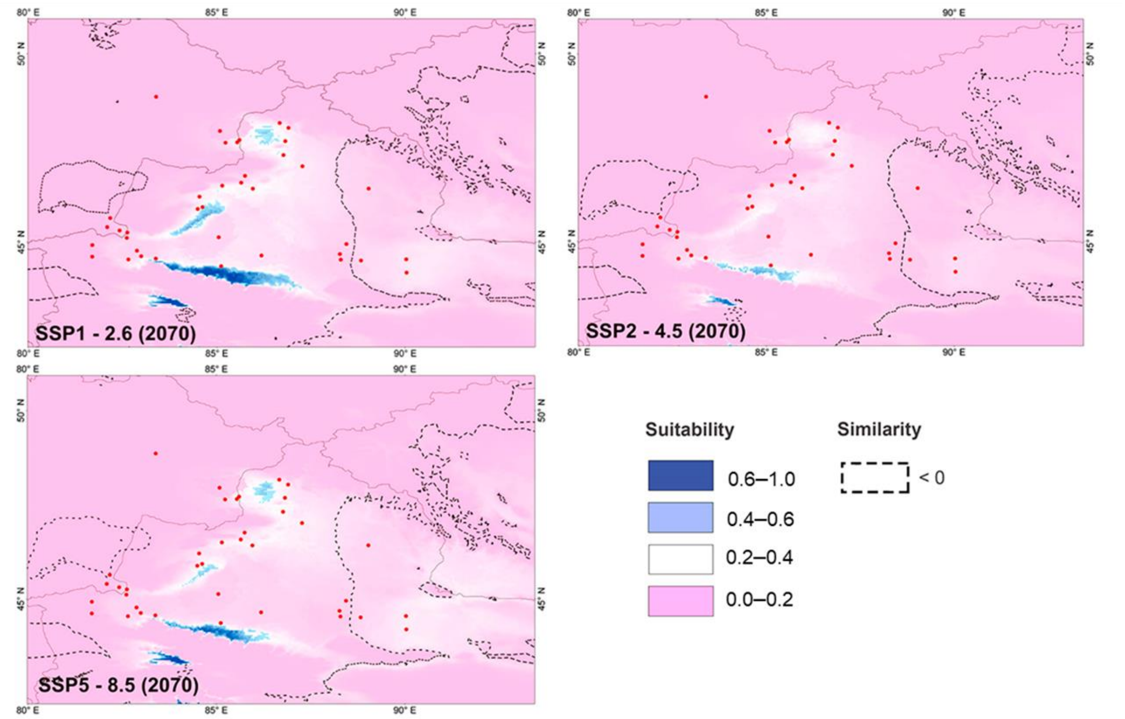

Figure S6. Potentially suitable distribution area for *P. melanurus* in 2070 (2061-2080) under three different gas emission models, Shared Socio-economic Pathway SSP1-2.6; SSP2-4.5; SSP5-8.5. Red dots indicate the locality of occurrence data.

Table S1. List of analyzed specimens, geographical origin, Clade assignment and GenBank accession number.

Site number	Voucher number	Haplotype number	Locality (Latitude/Longitude)	Clade/ Subclade	GenBank accession number
Site 1	Guo3161	H6	Ulungur (47.01/87.26)	IIb3	MW856942
Site 1	Guo3168	H6	Ulungur (47.01/87.26)	IIb3	MW856943
Site 1	Guo3169	H12	Ulungur (47.01/87.26)	IIb3	MW856944
Site 1	Guo3170	H6	Ulungur (47.01/87.26)	IIb3	MW856945
Site 1	Guo3171	H13	Ulungur (47.01/87.26)	IIb3	MW856946
Site 2	GXG294	H6	Karamay (46.41/85.95)	IIb3	MW857007
Site 2	GXG295	H6	Karamay (46.41/85.95)	IIb3	MW857008
Site 2	GXG296	H31	Karamay (46.41/85.95)	IIb3	MW857009
Site 2	GXG297	H13	Karamay (46.41/85.95)	IIb3	MW857010
Site 2	GXG298	H32	Karamay (46.41/85.95)	IIb3	MW857011
Site 2	GXG299	H33	Karamay (46.41/85.95)	IIb3	MW857012
Site 2	GXG303	H34	Karamay (46.41/85.95)	IIb3	MW857013
Site 2	GXG654	H31	Karamay (46.41/85.95)	IIb3	MW857058
Site 2	GXG655	H6	Karamay (46.41/85.95)	IIb3	MW857059
Site 3	Guo2320	H6	Karamay (45.13/85.05)	IIb3	MW856928
Site 3	Guo2321	H7	Karamay (45.13/85.05)	IIb3	MW856929
Site 3	Guo2322	H6	Karamay (45.13/85.05)	IIb3	MW856930
Site 3	Guo2324	H6	Karamay (45.13/85.05)	IIb3	MW856931
Site 4	GXG352	H41	Hoboksar (46.76/85.74)	IIb3	MW857029
Site 4	GXG353	H41	Hoboksar (46.76/85.74)	IIb3	MW857030
Site 4	GXG354	H13	Hoboksar (46.76/85.74)	IIb3	MW857031
Site 4	GXG355	H41	Hoboksar (46.76/85.74)	IIb3	MW857032
Site 4	GXG356	H13	Hoboksar (46.76/85.74)	IIb3	MW857033
Site 4	GXG357	H13	Hoboksar (46.76/85.74)	IIb3	MW857034
Site 4	GXG358	H41	Hoboksar (46.76/85.74)	IIb3	MW857035
Site 4	GXG359	H41	Hoboksar (46.76/85.74)	IIb3	MW857036
Site 4	GXG360	H13	Hoboksar (46.76/85.74)	IIb3	MW857037
Site 4	GXG665	H41	Hoboksar (46.76/85.74)	IIb3	MW857062
Site 4	GXG666	H41	Hoboksar (46.76/85.74)	IIb3	MW857063
Site 5	GXG315	H6	Hoboksar (46.57/85.64)	IIb3	MW857014
Site 5	GXG316	H35	Hoboksar (46.57/85.64)	IIb3	MW85701
Site 5	GXG318	H36	Hoboksar (46.57/85.64)	IIb3	MW857016
Site 5	GXG319	H36	Hoboksar (46.57/85.64)	IIb3	MW857017
Site 5	GXG320	H35	Hoboksar (46.57/85.64)	IIb3	MW857018
Site 5	GXG321	H35	Hoboksar (46.57/85.64)	IIb3	MW857019
Site 6	GXG325	H37	Hoboksar (46.50/85.14)	IIb3	MW857020
Site 6	GXG326	H6	Hoboksar (46.50/85.14)	IIb3	MW857021
Site 6	GXG327	H38	Hoboksar (46.50/85.14)	IIb3	MW857022
Site 6	GXG328	H38	Hoboksar (46.50/85.14)	IIb3	MW857023
Site 6	GXG329	H37	Hoboksar (46.50/85.14)	IIb3	MW857024
Site 6	GXG330	H39	Hoboksar (46.50/85.14)	IIb3	MW857025
Site 6	GXG331	H37	Hoboksar (46.50/85.14)	IIb3	MW857026
Site 6	GXG332	H6	Hoboksar (46.50/85.14)	IIb3	MW857027
Site 6	GXG657	H38	Hoboksar (46.50/85.14)	IIb3	MW857060
Site 6	GXG658	H47	Hoboksar (46.50/85.14)	IIb3	MW857061
Site 7	GXG351	H40	Emin (46.20/84.53)	IIb3	MW857028
Site 8	Guo2300	H4	Toli (45.88/84.49)	IIb3	MW856984
Site 8	Guo2301	H4	Toli (45.88/84.49)	IIb3	MW856926
Site 8	Guo2302	H5	Toli (45.88/84.49)	IIb3	MW856927

Site 9	GXG293	H4	Toli (45.93/84.62)	IIb3	MW857006
Site 9	GXG652	H4	Toli (45.93/84.62)	IIb3	MW857057
Site 10	GXG572	H6	Fuyun (46.42/89.02)	IIb3	MW857051
Site 10	GXG573	H6	Fuyun (46.42/89.02)	IIb3	MW857052
Site 10	GXG574	H6	Fuyun (46.42/89.02)	IIb3	MW857053
Site 10	GXG575	H6	Fuyun (46.42/89.02)	IIb3	MW857054
Site 10	GXG710	H6	Fuyun (46.42/89.02)	IIb3	MW857073
Site 10	GXG711	H6	Fuyun (46.42/89.02)	IIb3	MW857074
Site 11	GXG379	H6	Jeminay (47.31/86.76)	IIb3	MW857038
Site 11	GXG380	H6	Jeminay (47.31/86.76)	IIb3	MW857039
Site 11	GXG381	H42	Jeminay (47.31/86.76)	IIb3	MW857040
Site 11	GXG382	H43	Jeminay (47.31/86.76)	IIb3	MW857041
Site 11	GXG383	H44	Jeminay (47.31/86.76)	IIb3	MW857042
Site 11	GXG384	H45	Jeminay (47.31/86.76)	IIb3	MW857043
Site 11	GXG385	H38	Jeminay (47.31/86.76)	IIb3	MW857044
Site 11	GXG387	H6	Jeminay (47.31/86.76)	IIb3	MW857045
Site 11	GXG390	H46	Jeminay (47.31/86.76)	IIb3	MW857046
Site 11	GXG404	H6	Jeminay (47.31/86.76)	IIb3	MW857047
Site 11	GXG681	H6	Jeminay (47.31/86.76)	IIb3	MW857064
Site 11	GXG682	H48	Jeminay (47.31/86.76)	IIb3	MW857065
Site 11	GXG683	H6	Jeminay (47.31/86.76)	IIb3	MW857066
Site 12	Guo2208	H1	Shihezi (44.64/86.18)	IIb3	MW856918
Site 13	Guo4240	H16	Fukang (44.95/88.43)	IIb2	MW856953
Site 13	Guo4241	H17	Fukang (44.95/88.43)	IIb2	MW856954
Site 13	Guo4242	H18	Fukang (44.95/88.43)	IIb2	MW856955
Site 13	Guo4292	H17	Fukang (44.95/88.43)	IIb2	MW856961
Site 14	Guo4300	H21	Fukang (44.53/88.28)	IIb2	MW856962
Site 14	Guo4472	H21	Fukang (44.53/88.28)	IIb2	MW856963
Site 14	Guo4473	H17	Fukang (44.53/88.28)	IIb2	MW856964
Site 15	Guo4576	H21	Fukang (44.68/88.26)	IIb2	MW856966
Site 15	Guo4577	H21	Fukang (44.68/88.26)	IIb2	MW856967
Site 16	Guo4579	H22	Jimsar (44.51/88.82)	IIb2	MW856968
Site 16	Guo4580	H22	Jimsar (44.51/88.82)	IIb2	MW856969
Site 16	Guo4581	H23	Jimsar (44.51/88.82)	IIb2	MW856970
Site 16	Guo4286	H16	Jimsar (44.51/88.82)	IIb2	MW856960
Site 17	GXG255	H27	Ebinur (45.11/82.61)	IIb1	MW856994
Site 17	GXG256	H30	Ebinur (45.11/82.61)	IIb1	MW856995
Site 18	Guo7432	H25	Ebinur (44.77/82.87)	IIb1	MW856978
Site 19	Guo6433	H24	Bortala (44.61/81.69)	IIb1	MW856975
Site 19	Guo6434	H24	Bortala (44.61/81.69)	IIb1	MW856976
Site 19	Guo7437	H24	Bortala (44.61/81.69)	IIb1	MW856979
Site 19	Guo7438	H24	Bortala (44.61/81.69)	IIb1	MW856980
Site 19	Guo7439	H26	Bortala (44.61/81.69)	IIb1	MW856981
Site 19	Guo7440	H24	Bortala (44.61/81.69)	IIb1	MW856982
Site 19	Guo7441	H24	Bortala (44.61/81.69)	IIb1	MW856983
Site 20	GXG283	H3	Bortala (45.19/82.60)	IIb1	MW857005
Site 21	Guo4087	H14	Kuytun (44.36/85.11)	IIb1	MW856947
Site 22	GXG234	H27	Bole (44.92/81.69)	IIb1	MW856985
Site 22	GXG235	H28	Bole (44.92/81.69)	IIb1	MW856986
Site 22	GXG236	H24	Bole (44.92/81.69)	IIb1	MW856987
Site 22	GXG237	H28	Bole (44.92/81.69)	IIb1	MW856988
Site 22	GXG238	H24	Bole (44.92/81.69)	IIb1	MW856989
Site 22	GXG239	H24	Bole (44.92/81.69)	IIb1	MW856990
Site 22	GXG242	H27	Bole (44.92/81.69)	IIb1	MW856991
Site 22	GXG244	H24	Bole (44.92/81.69)	IIb1	MW856992
Site 22	GXG246	H29	Bole (44.92/81.69)	IIb1	MW856993

Site 22	GXG635	H27	Bole (44.92/81.69)	IIb1	MW857055
Site 22	GXG637	H27	Bole (44.92/81.69)	IIb1	MW857056
Site 23	Guo4562	H19	Qitai (44.19/90.03)	IIa	MW856965
Site 24	Guo4258	H2	Qitai (44.54/90.02)	IIb1	MW856956
Site 24	Guo4259	H19	Qitai (44.54/90.02)	IIa	MW856957
Site 24	Guo4260	H20	Qitai (44.54/90.02)	IIa	MW856958
Site 24	Guo4262	H19	Qitai (44.54/90.02)	IIa	MW856959
Site 25	GXG257	H3	Alashankou (45.25/82.62)	IIb1	MW856996
Site 25	GXG258	H3	Alashankou (45.25/82.62)	IIb1	MW856997
Site 25	GXG259	H30	Alashankou (45.25/82.62)	IIb1	MW856998
Site 25	GXG260	H27	Alashankou (45.25/82.62)	IIb1	MW856999
Site 25	GXG262	H3	Alashankou (45.25/82.62)	IIb1	MW857000
Site 25	GXG263	H3	Alashankou (45.25/82.62)	IIb1	MW857001
Site 25	GXG268	H30	Alashankou (45.25/82.62)	IIb1	MW857002
Site 25	GXG270	H3	Alashankou (45.25/82.62)	IIb1	MW857003
Site 25	GXG271	H3	Alashankou (45.25/82.62)	IIb1	MW857004
Site 26	Guo2209	H2	Jinghe (44.54/82.65)	IIb1	MW856919
Site 26	Guo2210	H2	Jinghe (44.54/82.65)	IIb1	MW856920
Site 26	Guo2211	H2	Jinghe (44.54/82.65)	IIb1	MW856921
Site 26	Guo2212	H2	Jinghe (44.54/82.65)	IIb1	MW856922
Site 26	Guo2213	H2	Jinghe (44.54/82.65)	IIb1	MW856923
Site 26	Guo2214	H2	Jinghe (44.54/82.65)	IIb1	MW856924
Site 26	Guo4095	H15	Jinghe (44.54/82.65)	IIb1	MW856948
Site 26	Guo4096	H2	Jinghe (44.54/82.65)	IIb1	MW856949
Site 26	Guo4100	H2	Jinghe (44.54/82.65)	IIb1	MW856950
Site 26	Guo4101	H2	Jinghe (44.54/82.65)	IIb1	MW856951
Site 26	Guo4102	H2	Jinghe (44.54/82.65)	IIb1	MW856952
Site 27	Guo541	H2	Jinghe (44.54/82.58)	IIb1	MW856971
Site 27	Guo542	H3	Jinghe (44.54/82.58)	IIb1	MW856972
Site 27	Guo544	H2	Jinghe (44.54/82.58)	IIb1	MW856973
Site 27	Guo545	H2	Jinghe (44.54/82.58)	IIb1	MW856974
Site 28	Guo7427	H3	Jinghe (44.56/83.37)	IIb1	MW856977
Site 29	Guo2285	H3	Jinghe (44.54/82.58)	IIb1	MW856925
Site 30	Guo3084	H11	Bolade (48.16/86.66)	I	MW856941
Site 31	Guo3064	H10	Buerjin (48.03/86.90)	I	MW856936
Site 31	Guo3065	H10	Buerjin (48.03/86.90)	I	MW856937
Site 31	Guo3066	H10	Buerjin (48.03/86.90)	I	MW856938
Site 31	Guo3067	H10	Buerjin (48.03/86.90)	I	MW856939
Site 31	Guo3068	H10	Buerjin (48.03/86.90)	I	MW856940
Site 32	GXG411	H9	Buerjin (47.68/86.81)	I	MW857048
Site 32	GXG412	H9	Buerjin (47.68/86.81)	I	MW857049
Site 32	GXG413	H9	Buerjin (47.68/86.81)	I	MW857050
Site 32	GXG692	H9	Buerjin (47.68/86.81)	I	MW857067
Site 32	GXG693	H9	Buerjin (47.68/86.81)	I	MW857068
Site 32	GXG694	H9	Buerjin (47.68/86.81)	I	MW857069
Site 32	GXG695	H9	Buerjin (47.68/86.81)	I	MW857070
Site 32	GXG696	H9	Buerjin (47.68/86.81)	I	MW857071
Site 32	GXG697	H9	Buerjin (47.68/86.81)	I	MW857072
Site 33	Guo3028	H8	Zaisan (47.71/85.59)	I	MW856932
Site 33	Guo3030	H8	Zaisan (47.71/85.59)	I	MW856933
Site 33	Guo3038	H9	Zaisan (47.71/85.59)	I	MW856934
Site 33	Guo3040	H9	Zaisan (47.71/85.59)	I	MW856935
Site 34	KZL118	H9	Kurchum (47.94/85.08)	I	MW857082
Site 34	KZL119	H9	Kurchum (47.94/85.08)	I	MW857078
Site 34	KZL120	H50	Kurchum (47.94/85.08)	I	MW857079
Site 34	KZL121	H9	Kurchum (47.94/85.08)	I	MW857080

Site 34	KZL122	H9	Kurchum (47.94/85.08)	I	MW857081
Site 35	KZ202	H49	Kurchum type locality (48.85/83.38)	I	MW857075
Site 36	KZL107	H49	Kokpekty district E KZ (48.80/83.42)	I	MW857076
Site 36	KZL108	H49	Kokpekty district E KZ (48.80/83.42)	I	MW857077
Sequences from previous studies					
Site 37	KF691725	H52	Bukhtarma (48.83/83.43)	I	[38]
Site 37	MK461427	H56	Bukhtarma (48.83/83.43)	I	[34]
Site 38	MK461428	H54	Alakol (45.64/82.17)	IIb1	[38]
Site 38	KF691726	H51	Alakol (45.64/82.17)	IIb1	[38]
Site 39	MF567976	H51	Dzungar Gate (45.31/82.42)	IIb1	[34]
Site 40	MK461426	H53	Zaisan county (47.65/85.54)	I	[34]
Site 41	MK461384	H55	Zhalanashkol Lake (45.40/82.09)	IIb1	[34]
Site 42	MK461456	H55	Dzungar Gate (45.23/82.48)	IIb1	[34]
Site 43	MK461378	H57	Black Irtysh (47.90/85.01)	I	[34]
Site 44	MK461377	H58	Aygyrkum (47.64/85.23)	I	[34]
**Sequences used in Bayesian divergence time estimation**					
	KF691727		*P. sp. 2*		[38]
	MK461395		*P. kuschakewitschii*		[34]
	MK461358		*P. kuschakewitschii*		[34]
	MK461359		*P. kuschakewitschii*		[34]
	MK461373		*P. guttatus*		[34]
	MK461409		*P. guttatus*		[34]
	MK461459		*P. incertus*		[34]
	MK461441		*P. incertus*		[34]
	MK461442		*P. incertus*		[34]
	MK461443		*P. incertus*		[34]
	MK461444		*P. incertus*		[34]
	MK461453		*P. incertus*		[34]
	MK461457		*P. incertus*		[34]
	MK461333		*P. alpherakii*		[34]
	MK461334		*P. alpherakii*		[34]
	MK461335		*P. alpherakii*		[34]
	MK461336		*P. alpherakii*		[34]
	MK461337		*P. alpherakii*		[34]
	MK461338		*P. alpherakii*		[34]
	MK461341		*P. alpherakii*		[34]
	MK461342		*P. alpherakii*		[34]
	MK461343		*P. alpherakii*		[34]
	KF691729		*P. alpherakii*		[38]
	KF691730		*P. moltschanovi*		[38]
	MK461455		*P. moltschanovi*		[34]
	MK461381		*P. g. guttatus*		[34]
	MK461383		*P. g. guttatus*		[34]
	MK461385		*P. g. guttatus*		[34]
	MK461386		*P. g. guttatus*		[34]
	MK461368		*P. g. guttatus*		[34]
	MK461369		*P. g. guttatus*		[34]
	MK461394		*P. g. guttatus*		[34]
	MK461469		*P. g. guttatus*		[34]
	MK461460		*P. g. salsatus*		[34]
	MK461454		*P. g. salsatus*		[34]
	KF691732		*P. hispidus*		[38]
	KF691738		*P. hispidus*		[38]

	MF288957		*P. versicolor*		Dunayev et al., unpublished
	KF691736		*P. versicolor*		[38]
	MF288956		*P. v. kulagini*		Dunayev et al., unpublished
	KF691735		*P. v. kulagini*		[38]
	KF691734		*P. przewalskii*		[38]
	KF691704		*P. interscapularis*		[38]
	KF691705		*P. interscapularis*		[38]

[34] Dunayev, E.A.; Solovyova, E.N.; Poyarkov, N.A. Taxonomy, phylogeny and distribution of *Phrynocephalus* (superspecies *guttatus*) (Reptilia: Agamidae). *Curr. Stud. Herpetol.* **2020**, *20*, 16–34. [In Russian]. https://doi.org/10.18500/1814-6090-2020-20-1-2-16-34

[38] Solovyeva, E.N.; Poyarkov, N.A.; Dunayev, E.A.; Nazarov, R.A.; Lebedev, V.S.; Bannikova, A.A. Phylogenetic relationships and subgeneric taxonomy of toad headed agamas *Phrynocephalus* (Reptilia, Squamata, Agamidae) as determined by mitochondrial DNA sequencing. *Dokl. Biol. Sci.* **2014**, *455*, 119–124. https://doi.org/10.1134/S0012496614020148

Table S2. Environmental variables highly contributed to the SDM of *P. melanurus* with Pearson correlation coefficients $r \geq 0.6$. St. dev., standard deviation.

**Environmental variable**	**Contribution rate ± st. dev.**	**Permutation importance**
Annual mean temperature (Bio1)	28.8 ±2.12	25.8
Annual precipitation (Bio12)	26.2 ±13.22	44.9
Temperature of the wettest quarter (Bio8)	18.3 ±5.44	10.6
Mean diurnal range of temperature (Bio2)	12 ±3.18	7.5
Isothermality (Bio3)	7.7 ±0.42	8.3
Precipitation Seasonality (Bio15)	7 ±2.89	2.9

Table S3. Maxent model parameterization ranking obtained from R package ENMeval. FC = Feature class, RM = Regularization multiplier, AUC = Area under the curve, OR10 = omission rate of testing localities at the 10% training threshold, AICc = Akaike`s Information Criterion.

	FC	RM	Tune.args	AUC train.	Cbi. Train.	AUC diff. avg.	AUC diff. sd.	AUC val. avg.	AUC val. sd.	Cbi. val. avg.	Cbi. val. sd.	OR. 10p. avg.	OR. 10p. sd	OR. mtp. avg.	OR. mtp. sd.	AICc	delta. AICc	w.AICc	ncoef
1	L	0.1	fc.L_rm.0.1	0.856	0.774	0.103	0.485	0.843	0.774	*NA*	*NA*	0.131	2.056	0.026	0.973	711.031	22.370	9.814	6
2	LQ	0.1	Fc.LQ_rm.0.1	0.919	0.926	0.078	0.563	0.891	0.705	*NA*	*NA*	0.157	2.218	0.026	0.973	688.660	**0.000**	7.072	12
3	H	0.1	fc.H_rm.0.1	0.949	0.884	0.099	0.630	0.876	0.737	*NA*	*NA*	0.394	2.973	0.105	1.866	NA	NA	NA	67
4	LQH	0.1	fc.LQH_rm.0.1	0.950	0.917	0.098	0.626	0.876	0.732	*NA*	*NA*	0.394	2.973	0.105	1.866	NA	NA	NA	64
5	LQHP	0.1	fc.LQHP_rm.0.1	0.952	0.868	0.100	0.675	0.876	0.772	*NA*	*NA*	0.421	3.003	0.078	1.640	NA	NA	NA	71
6	LQHPT	0.1	fc.LQHPT_rm.0.1	0.991	0.870	0.143	0.792	0.849	0.799	*NA*	*NA*	0.815	2.358	0.526	3.037	NA	NA	NA	104
7	L	0.5	fc.L_rm.0.5	0.856	0.761	0.103	0.482	0.844	0.769	*NA*	*NA*	0.105	1.866	0.026	0.973	711.060	22.399	9.672	6
8	LQ	0.5	fc.LQ_rm.0.5	0.910	0.959	0.074	0.528	0.889	0.666	*NA*	*NA*	0.157	2.218	0.026	0.973	690.806	2.145	2.418	10
9	H	0.5	fc.H_rm.0.5	0.934	0.855	0.080	0.470	0.898	0.617	*NA*	*NA*	0.263	2.678	0.078	1.640	1494.6109	805.950	6.912	34
10	LQH	0.5	fc.LQH_rm.0.5	0.932	0.895	0.079	0.505	0.895	0.651	*NA*	*NA*	0.263	2.678	0.078	1.640	759.389	70.728	3.097	23
11	LQHP	0.5	fc.LQHP_rm.0.5	0.934	0.866	0.085	0.507	0.894	0.647	*NA*	*NA*	0.263	2.678	0.078	1.640	773.625	84.964	2.509	24
12	LQHPT	0.5	fc.LQHPT_rm.0.5	0.945	0.950	0.102	0.528	0.891	0.656	*NA*	*NA*	0.315	2.827	0.105	1.866	1104.076	415.415	4.397	32
13	L	1	fc.L_rm.1	0.857	0.786	0.078	0.479	0.845	0.765	*NA*	*NA*	0.105	1.866	0.026	0.973	708.318	19.657	3.809	5
14	LQ	1	fc.LQ_rm.1	0.900	0.874	0.075	0.505	0.881	0.667	*NA*	*NA*	0.157	2.218	0.026	0.973	695.144	6.484	2.764	9
15	H	1	fc.H_rm.1	0.929	0.893	0.075	0.454	0.902	0.611	*NA*	*NA*	0.131	2.056	0.078	1.640	968.260	279.599	1.365	30
16	LQH	1	fc.LQH_rm.1	0.927	0.906	0.076	0.505	0.897	0.642	*NA*	*NA*	0.131	2.056	0.052	1.358	722.912	34.251	2.581	19
17	LQHP	1	fc.LQHP_rm.1	0.928	0.933	0.078	0.505	0.897	0.649	*NA*	*NA*	0.131	2.056	0.052	1.358	742.755	54.094	1.267	21
18	LQHPT	1	fc.LQHPT_rm.1	0.935	0.932	0.102	0.491	0.897	0.636	*NA*	*NA*	0.210	2.479	0.078	1.640	739.953	105.293	9.668	25
19	L	1.5	fc.L_rm.1.5	0.856	0.782	0.083	0.481	0.845	0.767	*NA*	*NA*	0.131	2.056	0.026	1.358	708.421	19.761	3.617	5
20	LQ	1.5	fc.LQ_rm.1.5	0.887	0.778	0.074	0.485	0.869	0.677	*NA*	*NA*	0.131	2.056	0.078	1.640	703.798	15.138	3.650	9
21	H	1.5	fc.H_rm.1.5	0.926	0.922	0.077	0.469	0.900	0.618	*NA*	*NA*	0.131	2.056	0.052	1.358	855.182	166.521	4.895	27
22	LQH	1.5	fc.LQH_rm.1.5	0.921	0.914	0.077	0.521	0.890	0.661	*NA*	*NA*	0.157	2.218	0.052	1.358	708.442	19.781	3.580	16
23	LQHP	1.5	fc.LQHP_rm.1.5	0.923	0.953	0.078	0.532	0.894	0.674	*NA*	*NA*	0.131	2.056	0.078	1.640	702.852	14.191	5.859	15
24	LQHPT	1.5	fc.LQHPT_rm.1.5	0.931	0.960	0.103	0.480	0.897	0.632	*NA*	*NA*	0.184	2.358	0.026	0.973	708.114	19.454	4.217	17
25	L	2	fc.L_rm.2	0.856	0.793	0.084	0.486	0.845	0.771	*NA*	*NA*	0.131	2.056	0.052	1.358	708.571	19.911	3.356	5
26	LQ	2	fc.LQ_rm.2	0.879	0.760	0.074	0.454	0.867	0.665	*NA*	*NA*	0.131	2.056	0.078	1.640	699.505	10.844	3.123	6
27	H	2	fc.H_rm.2	0.921	0.921	0.081	0.475	0.895	0.623	*NA*	*NA*	0.184	2.358	0.052	1.358	797.949	109.288	1.311	24
28	LQH	2	fc.LQH_rm.2	0.913	0.824	0.081	0.508	0.882	0.670	*NA*	*NA*	0.157	2.218	0.052	1.358	709.939	21.279	1.693	15
29	LQHP	2	fc.LQHP_rm.2	0.917	0.913	0.082	0.544	0.886	0.695	*NA*	*NA*	0.157	2.218	0.078	1.640	716.486	27.825	6.416	16
30	LQHPT	2	fc.LQHPT_rm.2	0.926	0.965	0.103	0.493	0.889	0.653	*NA*	*NA*	0.210	2.479	0.026	0.973	709.402	20.741	2.215	16
31	L	2.5	fc.L_rm.2.5	0.856	0.788	0.086	0.489	0.844	0.774	*NA*	*NA*	0.131	2.056	0.052	1.358	708.766	20.105	3.045	5
32	LQ	2.5	fc.LQ_rm.2.5	0.876	0.788	0.074	0.448	0.864	0.674	*NA*	*NA*	0.157	2.218	0.078	1.640	701.499	12.838	1.152	6
33	H	2.5	fc.H_rm.2.5	0.916	0.880	0.083	0.486	0.889	0.633	*NA*	*NA*	0.184	2.358	0.052	1.358	790.323	101.662	5.939	23
34	LQH	2.5	fc.LQH_rm.2.5	0.905	0.771	0.078	0.492	0.879	0.671	*NA*	*NA*	0.157	2.218	0.052	1.358	705.190	16.529	1.820	13
35	LQHP	2.5	fc.LQHP_rm.2.5	0.912	0.848	0.083	0.531	0.886	0.679	*NA*	*NA*	0.184	2.358	0.078	1.640	701.723	13.063	1.030	12
36	LQHPT	2.5	fc.LQHPT_rm.2.5	0.918	0.919	0.104	0.513	0.883	0.672	*NA*	*NA*	0.210	2.479	0.026	0.973	706.961	18.301	7.507	14

37	L	3	fc.L_rm.3	0.856	0.802	0.089	0.491	0.843	0.779	*NA*	*NA*	0.131	2.056	0.052	1.358	709.004	20.343	2.703	5
38	LQ	3	fc.LQ_rm.3	0.874	0.786	0.077	0.450	0.863	0.684	*NA*	*NA*	0.131	2.056	0.052	1.358	700.358	11.697	2.038	5
39	H	3	fc.H_rm.3	0.911	0.905	0.083	0.500	0.882	0.652	*NA*	*NA*	0.157	2.218	0.052	1.358	753.505	64.845	5.869	19
40	LQH	3	fc.LQH_rm.3	0.900	0.706	0.079	0.469	0.878	0.664	*NA*	*NA*	0.157	2.218	0.052	1.358	705.789	17.128	1.349	12
41	LQHP	3	fc.LQHP_rm.3	0.909	0.803	0.083	0.524	0.883	0.676	*NA*	*NA*	0.157	2.218	0.052	1.358	706.947	18.287	7.560	12
42	LQHPT	3	fc.LQHPT_rm.3	0.912	0.817	0.079	0.527	0.878	0.684	*NA*	*NA*	0.184	2.358	0.078	1.640	719.970	31.309	1.123	15
43	L	3.5	fc.L_rm.3.5	0.856	0.819	0.083	0.493	0.843	0.783	*NA*	*NA*	0.131	2.056	0.026	0.973	712.121	23.460	5.690	6
44	LQ	3.5	fc.LQ_rm.3.5	0.873	0.793	0.104	0.450	0.862	0.687	*NA*	*NA*	0.131	2.056	0.052	1.358	700.816	12.156	1.621	5
45	H	3.5	fc.H_rm.3.5	0.904	0.900	0.090	0.543	0.875	0.701	*NA*	*NA*	0.157	2.218	0.026	0.973	806.375	117.715	1.941	23
46	LQH	3.5	fc.LQH_rm.3.5	0.896	0.658	0.081	0.453	0.876	0.659	*NA*	*NA*	0.184	2.358	0.052	1.358	706.081	17.420	1.165	11
47	LQHP	3.5	fc.LQHP_rm.3.5	0.904	0.783	0.084	0.523	0.877	0.678	*NA*	*NA*	0.157	2.218	0.052	1.358	712.940	24.280	3.776	12
48	LQHPT	3.5	fc.LQHPT_rm.3.5	0.906	0.750	0.080	0.536	0.874	0.694	*NA*	*NA*	0.184	2.358	0.052	1.358	712.053	23.393	5.885	12
49	L	4	fc.L_rm.4	0.856	0.837	0.083	0.495	0.842	0.787	*NA*	*NA*	0.131	2.056	0.026	0.973	712.457	23.796	4.809	6
50	LQ	4	fc.LQ_rm.4	0.873	0.809	0.105	0.450	0.862	0.690	*NA*	*NA*	0.131	2.056	0.052	1.358	701.332	12.672	1.252	5
51	H	4	fc.H_rm.4	0.898	0.882	0.090	0.554	0.872	0.719	*NA*	*NA*	0.157	2.218	0.026	0.973	752.398	63.738	1.020	17
52	LQH	4	fc.LQH_rm.4	0.892	0.610	0.083	0.442	0.874	0.652	*NA*	*NA*	0.184	2.358	0.052	1.358	699.472	10.812	3.174	8
53	LQHP	4	fc.LQHP_rm.4	0.900	0.754	0.084	0.529	0.871	0.689	*NA*	*NA*	0.157	2.218	0.052	1.358	710.622	21.962	1.203	10
54	LQHPT	4	fc.LQHPT_rm.4	0.900	0.790	0.081	0.538	0.869	0.704	*NA*	*NA*	0.184	2.358	0.052	1.358	714.945	26.285	1.386	11
55	L	4.5	fc.L_rm.4.5	0.856	0.854	0.085	0.497	0.841	0.791	*NA*	*NA*	0.131	2.056	0.026	0.973	712.834	24.173	3.983	6
56	LQ	4.5	fc.LQ_rm.4.5	0.873	0.825	0.106	0.452	0.861	0.694	*NA*	*NA*	0.131	2.056	0.052	1.358	701.903	13.243	9.415	5
57	H	4.5	fc.H_rm.4.5	0.895	0.917	0.091	0.566	0.870	0.744	*NA*	*NA*	0.157	2.218	0.026	0.973	803.740	115.079	7.248	22
58	LQH	4.5	fc.LQH_rm.4.5	0.889	0.696	0.086	0.433	0.872	0.653	*NA*	*NA*	0.184	2.358	0.052	1.358	703.143	14.482	5.065	8
59	LQHP	4.5	fc.QHPL_rm.4.5	0.895	0.807	0.082	0.520	0.870	0.688	*NA*	*NA*	0.157	2.218	0.052	1.358	711.708	23.048	6.993	9
60	LQHPT	4.5	fc.LQHPT_rm.4.5	0.895	0.814	0.084	0.524	0.868	0.695	*NA*	*NA*	0.184	2.358	0.052	1.358	711.728	23.067	6.925	9
61	L	5	fc.L_rm.5	0.856	0.862	0.106	0.499	0.840	0.795	*NA*	*NA*	0.131	2.056	0.026	0.973	713.249	24.588	3.237	6
62	LQ	5	fc.LQ_rm.5	0.872	0.832	0.092	0.452	0.860	0.698	*NA*	*NA*	0.157	2.218	0.052	1.358	702.525	13.865	6.898	5
63	H	5	fc.H_rm.5	0.891	0.921	0.090	0.599	0.864	0.786	*NA*	*NA*	0.157	2.218	0.026	0.973	769.096	80.435	2.416	18
64	LQH	5	fc.LQH_rm.5	0.886	0.726	0.086	0.428	0.871	0.656	*NA*	*NA*	0.157	2.218	0.052	1.358	699.360	10.699	3.358	6
65	LQHP	5	fc.LQHP_rm.5	0.893	0.857	0.083	0.514	0.868	0.692	*NA*	*NA*	0.157	2.218	0.052	1.358	719.093	30.432	1.742	10
66	LQHPT	5	fc.LQHPT_rm.5	0.892	0.854	0.084	0.514	0.867	0.693	*NA*	*NA*	0.157	2.218	0.052	1.358	715.351	26.691	1.131	9
67	L	5.5	fc.L_rm.5.5	0.856	0.872	0.107	0.501	0.840	0.798	*NA*	*NA*	0.131	2.056	0.026	0.973	713.701	25.041	2.581	6
68	LQ	5.5	fc.LQ_rm.5.5	0.872	0.845	0.093	0.455	0.859	0.703	*NA*	*NA*	0.157	2.218	0.052	1.358	703.197	14.537	4.930	5
69	H	5.5	fc.H_rm.5.5	0.887	0.942	0.097	0.653	0.855	0.848	*NA*	*NA*	0.131	2.056	0.026	0.973	760.140	71.497	2.127	16
70	LQH	5.5	fc.LQH_rm.5.5	0.883	0.727	0.088	0.437	0.868	0.671	*NA*	*NA*	0.184	2.358	0.052	1.358	701.406	12.745	1.207	6
71	LQHP	5.5	fc.LQHP_rm.5.5	0.889	0.890	0.086	0.521	0.864	0.707	*NA*	*NA*	0.131	2.056	0.052	1.358	722.724	34.063	2.835	10
72	LQHPT	5.5	fc.LQHPT_rm.5.5	0.856	0.893	0.086	0.522	0.864	0.709	*NA*	*NA*	0.131	2.056	0.052	1.358	722.721	34.063	2.840	10
73	L	6	fc.L_rm.6	0.872	0.884	0.108	0.504	0.839	0.803	*NA*	*NA*	0.131	2.056	0.026	0.973	714.189	25.528	2.023	6
74	LQ	6	fc.LQ_rm.6	0.878	0.835	0.093	0.456	0.858	0.702	*NA*	*NA*	0.157	2.218	0.052	1.358	703.916	15.255	3.441	5
75	H	6	fc.H_rm.6	0.880	0.954	0.103	0.686	0.848	0.893	*NA*	*NA*	0.131	2.056	0.026	0.973	759.996	71.336	2.286	15
76	LQH	6	fc.LQHP_rm.6	0.880	0.770	0.090	0.445	0.863	0.688	*NA*	*NA*	0.157	2.218	0.052	1.358	703.526	14.865	4.182	6
77	LQHP	6	fc.LQHP_rm.6	0.885	0.924	0.089	0.526	0.860	0.723	*NA*	*NA*	0.131	2.2056	0.078	1.640	719.340	30.679	1.540	8
78	LQHPT	6	fc.LQHPT_rm.6	0.884	0.920	0.089	0.525	0.860	0.723	*NA*	*NA*	0.131	2.056	0.078	1.640	719.372	30.711	1.515	8

Table S4. Sampling sites retained after rarefication for species distribution modeling.

No.	Site	Sampling Site	Longitude	Latitude
1	Site 1	Ulungur	87.26	47.01
2	Site 2	Karamay	85.95	46.41
3	Site 3	Karamay	85.05	45.13
4	Site 4	Hoboksar	85.74	46.76
5	Site 5	Hoboksar	86.64	46.57
6	Site 6	Hoboksar	85.14	46.50
7	Site 7	Emin	84.53	46.20
8	Site 8	Toli	84.49	45.88
9	Site 9	Toli	84.62	45.93
10	Site 10	Fuyun	89.02	46.42
11	Site 11	Jeminay	86.76	47.31
12	Site 12	Shihezi	86.18	44.64
13	Site 13	Fukang	88.43	44.95
14	Site 14	Fukang	88.28	44.53
15	Site 15	Fukang	88.26	44.68
16	Site 16	Jimsar	88.82	44.51
17	Site 17	Ebinur	82.61	45.11
18	Site 18	Ebinur	82.87	44.77
19	Site 19	Bortala	81.69	44.61
20	Site 21	Kuytun	85.11	44.36
21	Site 22	Bole	81.69	44.92
22	Site 23	Qitai	90.03	44.19
23	Site 24	Qitai	90.02	44.54
24	Site 25	Alashankou	82.62	45.25
25	Site 26	Jinghe	82.65	44.54
26	Site 28	Jinghe	82.99	44.62
27	Site 29	Jinghe	83.37	44.56
28	Site 30	Bolade	86.66	48.16
29	Site 31	Buerjin	86.90	48.03
30	Site 32	Buerjin	86.81	47.68
31	Site 33	Zaisan	85.59	47.71
32	Site 34	Kurchum	85.08	47.94
33	Site 35	Kurchum	83.38	48.85
34	Site 38	Alakol	82.17	45.64
35	Site 39	Dzungar Gate	82.42	45.31
36	Site 40	Zaisan	85.54	47.65
37	Site 41	Zhalanashkol	82.09	45.40
38	Site 44	Aygyrkum	85.23	47.64

Table S5. Hierarchical analysis of AMOVA for testing the genetic subdivision of populations of *P. melanurus* using *COI* sequences. Statistical significance at $p \leq 0.001$.

**Source of variation**	***d.f.***	**Sum of squares**	**Variance components**	**% of variation**	**Fixation indices (*p-value*)**
2 groups (Zaisan Basin, Dzungar Basin)					
Among groups	1	322.178	7.00170	73.94	0.73936 (p = 0.000)
Among populations within groups	33	253.319	1.59203	16.81	0.64502 (p = 0.000)
Within populations	119	104.263	0.87616	9.25	0.90748 (p = 0.000)
Total	153	679.760	9.46989		
4 groups (Zaisan Basin, Central, West, and East of Dzungar Basin)					
Among groups	3	460.058	4.23897	73.11	0.73105 (p = 0.000)
Among populations within groups	31	115.439	0.68332	11.78	0.43817 (p = 0.000)
Within populations	119	104.263	0.87616	15.12	0.84890 (p = 0.000)
Total	153	679.760	5.79845		
